# Structural and Theoretical Assessment of Covalency in a Pu(III) Borohydride Complex

**DOI:** 10.1021/jacs.4c09888

**Published:** 2024-09-16

**Authors:** Joshua C. Zgrabik, Daniel J. Lussier, Rina Bhowmick, Ngan Nguyen, Peter A. Zacher, Tatyana Elkin, Andrew J. Gaunt, George S. Goff, Harris E. Mason, Jesse Murillo, Brian L. Scott, Bess Vlaisavljevich, Scott R. Daly

**Affiliations:** † Department of Chemistry, University of Iowa, E331 Chemistry Building, Iowa City, Iowa 52242, United States; ⊥ Los Alamos National Laboratory, P.O. Box 1663, Los Alamos, New Mexico 87545, United States; § University of South Dakota, 414 E. Clark St, Vermillion South Dakota 57069, United States

## Abstract

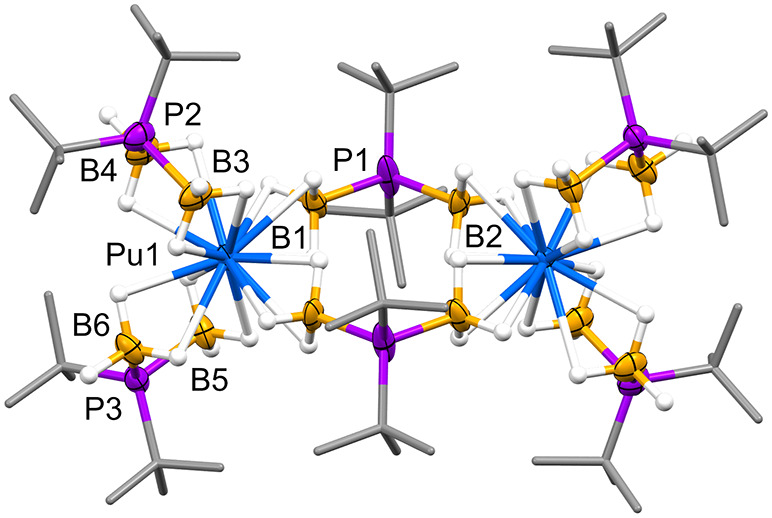

Despite the discovery of actinide borohydride complexes over 80 years ago, no plutonium borohydride complexes have been structurally validated using single-crystal X-ray diffraction (XRD). Here we describe Pu_2_(H_3_BP^*t*^Bu_2_BH_3_)_6_, the first example of a Pu(III) borohydride complex authenticated by XRD and NMR spectroscopy. Theoretical calculations (DFT, EDA, and QTAIM) and experimental comparisons of metal–boron distances suggest that metal–borohydride covalency in M_2_(H_3_BP^*t*^Bu_2_BH_3_)_6_ complexes generally decreases in the order M = U(III) > Pu(III) > Ln(III).

Actinide borohydrides, complexes containing An–H–B bonds, were discovered during the Manhattan Project when volatile U(BH_4_)_4_ was investigated for isotopically enriching uranium in U-235.^1,2^ The first crystal structure of an actinide borohydride, again U(BH_4_)_4_, was reported several decades later.^3,4^ These data revealed for the first time the defining structural characteristics of these complexes with uranium coordinated exclusively by hydrogen atoms.^5^

Though many structurally determined Th and U borohydride complexes have been identified since the discovery of U(BH_4_)_4_,^5−7^ there are very few examples beyond uranium. Np(BH_4_)_4_ and Np(MeBH_3_)_4_ are the only two transuranium borohydride complexes to be characterized by single-crystal X-ray diffraction (Figure 1).^8−14^ Pu(BH_4_)_4_, the only known plutonium borohydride complex,^15^ is an unstable liquid at room temperature.^11^ Powder X-ray diffraction (XRD) data showed that Pu(BH_4_)_4_ crystallizes in the same space group and has similar unit cell parameters as Np(BH_4_)_4_,^11,16^ but no data indicating atomic positions were reported.

**Figure 1:**
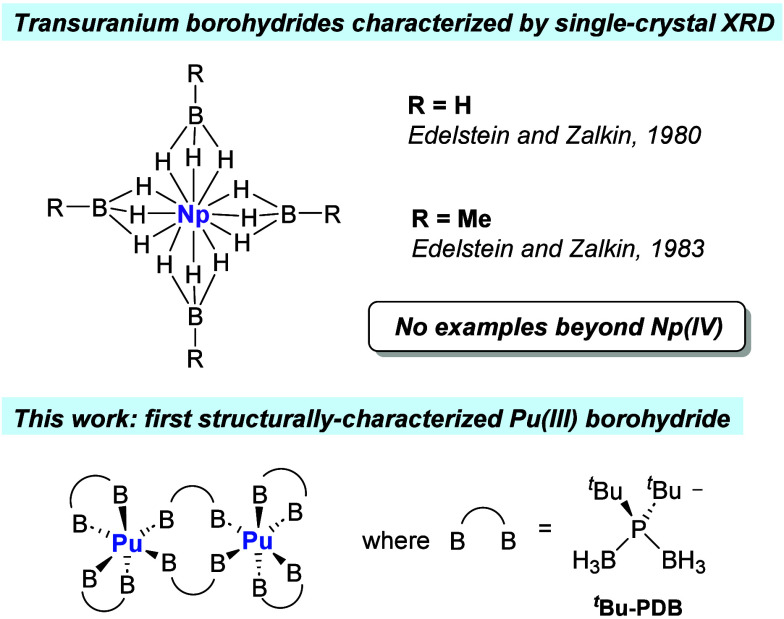
Transuranium borohydride complexes structurally characterized by single-crystal XRD.

The dearth of transuranium borohydride structures has come into focus recently because there has been growing evidence of how metal–borohydride covalency can influence the structures and properties of trivalent actinide complexes, a phenomenon commonly associated with more conventional soft donor ligands.^17−19^ These effects have been observed in U(III) borohydride complexes,^20−22^ but little is known about how they manifest as the 5f-block is traversed into the transuranium elemental realm.

One set of difficulties in preparing molecular borohydride complexes with Pu concerns radiological safety and isotope availability limitations, as well as the known pyrophoricity of borohydrides when complexed with actinides.^10,11,23−25^ Another challenge is the instability of Pu(IV) in the presence of reducing borohydride ligands. The aforementioned Pu(BH_4_)_4_, for example, decomposes via reduction to form Pu(III) products that have yet to be characterized.^11,16^ Once reduced, traditional borohydrides like BH_4_^1–^ and BH_3_Me^1–^ are too small to saturate the relatively large coordination sphere of Pu(III) to form homoleptic complexes soluble in organic solvents.^5,7^ Thus, the investigation of homoleptic Pu(III) borohydride complexes requires the development of borohydride ligands that can saturate the coordination sphere of this relatively large trivalent ion.^26^

We recently demonstrated how a class of larger borohydride ligands called phosphinodiboranates, which have the general formula H_3_BPR_2_BH_3_^1–^, can be used to prepare U_2_(H_3_BP^*t*^Bu_2_BH_3_)_6_ and Ln_2_(H_3_BP^*t*^Bu_2_BH_3_)_6_.^22,27−30^ These dinuclear complexes are isostructural regardless of metal size, which permitted structural comparisons that revealed shorter than expected U–B distances.^22^ Subsequent calculations corroborated the structural observations and converged to suggest that the uranium–borohydride bonds have increased covalency compared to those with lanthanides.^22^ Aside from its larger size, H_3_BP^*t*^Bu_2_BH_3_^1–^ was an ideal choice for extension to transuranium elements (that pose greater radiological hazards) because the U and Ln complexes have shown no appreciable volatility and they appear less susceptible to enflaming in air.^28^

Herein, we report how H_3_BP^*t*^Bu_2_BH_3_^1–^ (^*t*^Bu-PDB) was used to prepare the first Pu borohydride complex to be characterized by single-crystal XRD and NMR spectroscopy. Given the logistical and safety constraints associated with synthetic Pu chemistry in a fundamental research laboratory setting, we first had to develop procedures to prepare and crystallize the Pu complex on milligram scales. Using 9.0 mg of UI_3_(THF)_4_^24,31,32^ as a test surrogate, we showed that reactions with 6.3 mg of K(H_3_BP^*t*^Bu_2_BH_3_)^33^ in chlorobenzene, followed by crystallization from pentane, afforded red crystals of U_2_(H_3_BP^*t*^Bu_2_BH_3_)_6_ (**1**) in yields as high as 79% (5.5 mg; Figure 2). Chlorobenzene, a solvent shown by Edelstein and co-workers to be compatible with the synthesis of actinide borohydrides,^10^ was used because we previously showed that metathesis reactions with several phosphinodiboranate salts are low yielding and often irreproducible in Et_2_O and THF.^27,28^ Moreover, mechanochemical methods used to prepare other ^*t*^Bu-PDB complexes^22,29,30^ were not amenable to reactions with powdered Pu salts because of the contamination risk.

**Figure 2:**
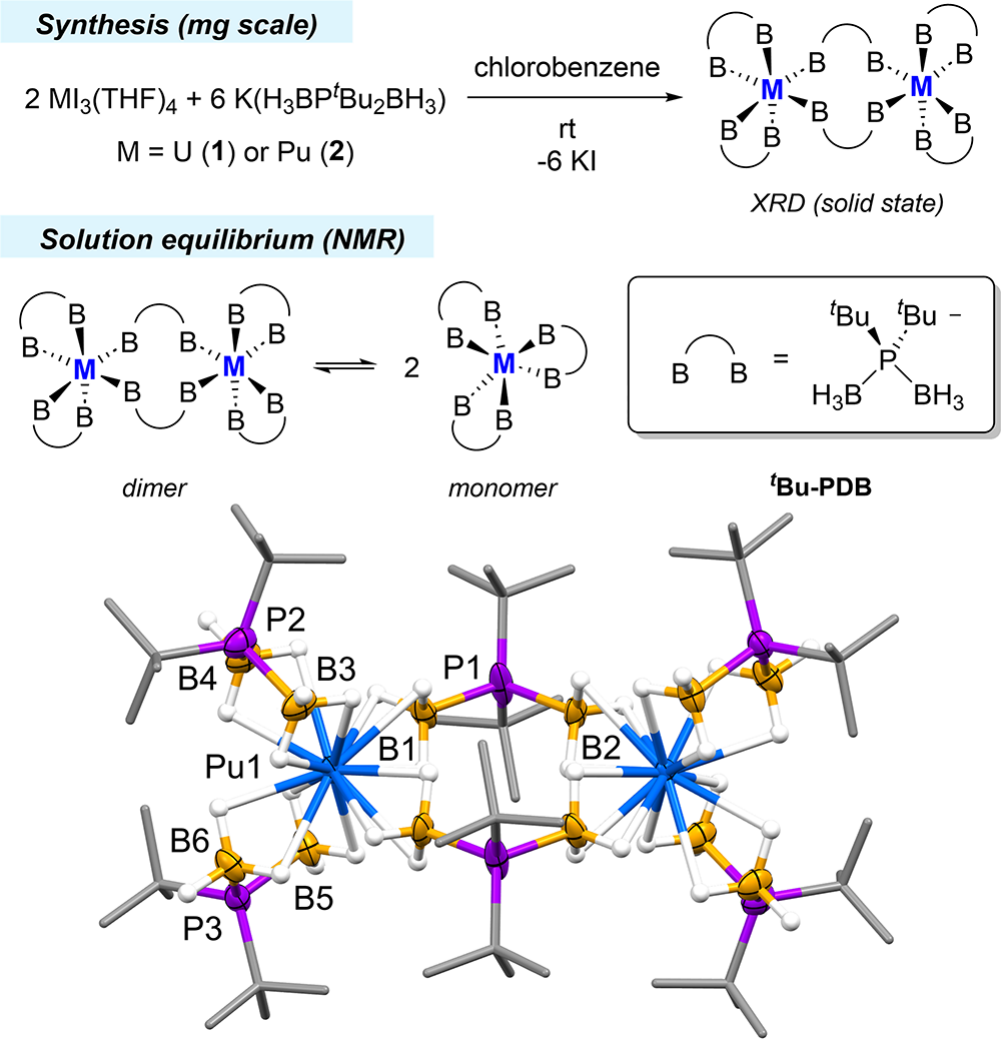
*Top* – Synthesis of M_2_(H_3_BP^*t*^Bu_2_BH_3_)_6_ and comparison of solution and solid-state structures for M = U (**1**) and Pu (**2**). *Bottom* – Molecular structure of **2**. Ellipsoids are drawn at 50%. Carbon atoms are shown as capped sticks, and hydrogen atoms attached to carbon were omitted from the figure.

Repeating the same mg-scale procedure using PuI_3_(THF)_4_ instead of UI_3_(THF)_4_ reproducibly afforded blue crystals of Pu_2_(H_3_BP^*t*^Bu_2_BH_3_)_6_ (**2**), as confirmed by single-crystal XRD (Figure 2). The structure is dinuclear and isostructural with **1** and homoleptic lanthanide ^*t*^Bu-PDB complexes reported previously.^22^ The structure has two chelating ligands per metal and two bridging ligands that form the dinuclear core. The metals are coordinated exclusively by hydrogen atoms and are tentatively assigned coordination numbers of 14 based on the Pu–B distances, but this may be lower, as suggested by DFT calculations (*vide infra*). The chelating Pu–B distances range from 2.848(6) to 2.950(6) Å, indicative of κ^2^-BH_3_ groups, whereas the bridging Pu–B distances are shorter at 2.675(6) and 2.678(5) Å and more consistent with κ^3^-BH_3_.

The experimental M–B distances in **2** were compared to those in **1** and isostructural lanthanide ^*t*^Bu-PDB complexes (Figure 3).^22^ Structural assessments of **1** revealed that bridging U–B distances were 0.04 Å shorter than expected when compared to the linear regression afforded by plotting Ln–B distances against Ln ionic radii, whereas the bridging Pu–B distances were 0.02 Å shorter.^34^ This suggests that the Pu–B bonds are less covalent than U–B bonds, which is consistent with systematic metal–ligand bond comparison studies of trivalent actinide complexes containing ligands with soft chalcogen donor groups.^35−37^

**Figure 3:**
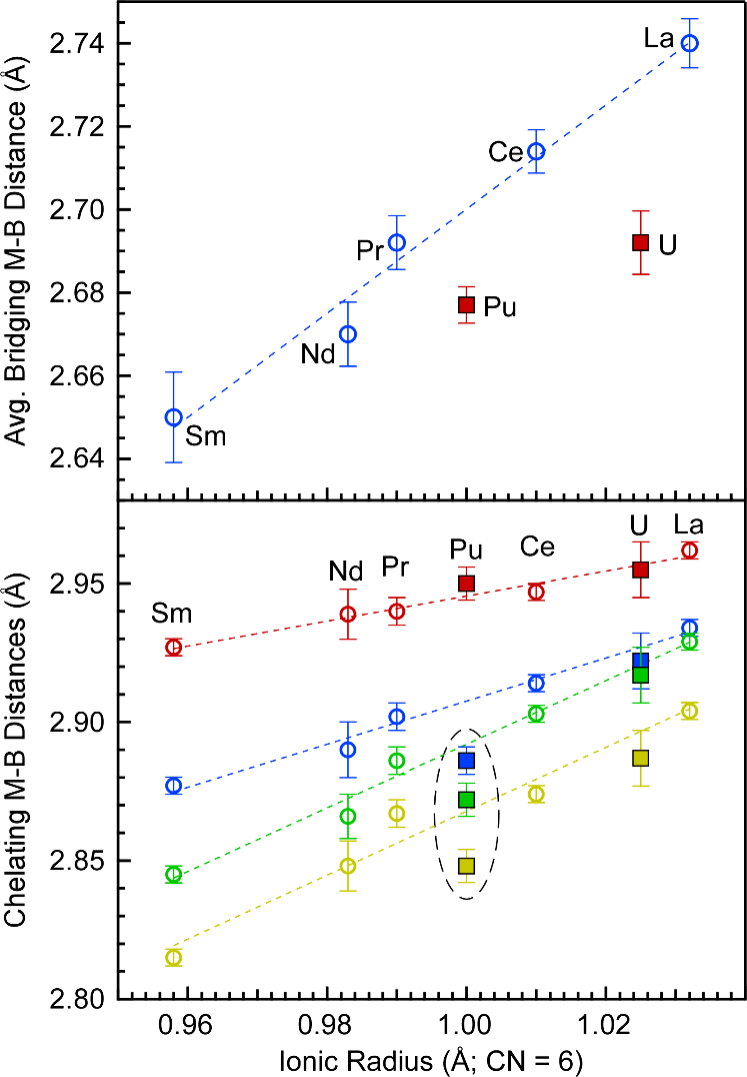
*Top* – Plot of average bridging M–B distances vs ionic radius^26^ of the metal for M_2_(H_3_BP^*t*^Bu_2_BH_3_)_6_ complexes (M = actinide or lanthanide). The error bars account for the standard deviation of the averaged M–B distances and the esd’s for the individual M–B distances (see SI for details). *Bottom* – Plot of chelating M–B distances vs ionic radius of the metal. The shorter chelating Pu–B distances are circled for emphasis. Actinides are represented by solid squares, whereas lanthanides are represented by open circles. The error bars represent esd values from XRD. Dashed lines in both plots represent linear regressions of the lanthanide data points. *R*^2^ values are all >0.96.

We next evaluated the chelating M–B distances. It was observed previously that the chelating U–B bonds in **1** show no significant departure from linear regressions obtained when plotting chelating Ln–B distances against their ionic radii (Figure 3). In contrast, data collected for **2** showed that the three shortest chelating Pu–B distances are all 0.02 Å shorter than expected. Only the longest (and presumably most ionic) Pu–B distance at 2.950(6) Å falls close to its respective line.

Before detailing calculations used to assess metal–ligand covalency in **2**, we describe its NMR data, the first for a Pu borohydride complex (see SI for solid-state UV–vis–NIR data for **2**). ^1^H, ^11^B, and ^31^P NMR data collected on crystals of **2** dissolved in C_6_D_6_ revealed that **2** deoligomerizes to form an equilibrium mixture of the dimer and the putative monomer Pu(H_3_BP^*t*^Bu_2_BH_3_)_3_ (**2a**), as described previously for other M_2_(H_3_BP^*t*^Bu_2_BH_3_)_6_ complexes.^22^ Three paramagnetically shifted and broadened ^11^B resonances were observed at δ −0.6, 9.7, and 44.7 ppm, and these complemented three ^31^P{^1^H} NMR resonances at δ −208.7, −159.2, and 158.9 ppm. Two of the resonances in each set are assigned to the dimer **2** (bridging and chelating ^*t*^Bu-PDB environments), and one of the resonances is assigned to the monomer **2a** (chelating ^*t*^Bu-PDB only). Consistent with the ^11^B and ^31^P data, the ^1^H NMR spectrum revealed three major ^*t*^Bu resonances at δ 0.88, 1.01, and 1.26 ppm. Additional ^1^H resonances assigned to the BH_3_ groups were more paramagnetically shifted due to their direct binding to Pu(III). A broad multiplet assigned to a single BH_3_ resonance was observed at δ 12.0 ppm, and overlapping resonances were observed at δ 20.9 ppm, similar to those reported with the lanthanide congener Sm.^22^

DFT calculations were performed to quantify the thermodynamics of the **2**/**2a** equilibrium for comparison to **1**. The structures of **2** and **2a** were calculated at the TPSS-D3/def2-TZVP, def-TZVP level of theory,^38,39^ as previously used for **1**.^22^ The optimized structure of **2** was in good agreement with the experimental data (see SI for details), and bond distances and angles for the calculated structures of **2** and **2a** are provided in Tables S1–S3. The Δ*G* for **1** and **2** are identical within error at 6.3 and 6.7 kcal·mol^–1^ and ∼2 kcal·mol^–1^ higher than those for the lanthanide complexes (Table S4). The increase in Δ*G* of deoligomerization for **1** and **2** is enthalpic in origin, suggesting it is attributable to stronger bridging actinide–borohydride bonds compared to the analogous lanthanide complexes.

Subsequent energy decomposition analysis (EDA) calculations were performed at the PBE/TZP level of theory to determine if the shortened Pu–B distances reflect increased covalency with the bridging and chelating ^*t*^Bu-PDB ligands. Starting with the bridging metal–ligand bonds, the orbitalic energy contribution for the trivalent lanthanide complexes was on average 35.8 ± 0.4% (Table S11). Consistent with the bridging M–B distances, this value was largest in **1** at 39.1%, but decreased to 36.6% in **2**. The orbitalic contribution for the chelating ligands in the lanthanide complexes was on average 36.7 ± 1.1% with larger values again obtained for **1** and **2** at 39.1% and 38.0%, respectively (Table S10). While the orbitalic contribution for Pu is closer to the lanthanides (Figure S22), there is a clear difference in the total interaction energies (Figure S23). The actinide complexes had interaction energies that were stronger for the bridging ligands, consistent with the aforementioned deoligomerization energies, but weaker compared to the lanthanides for the chelating ligands. Bond order calculations (PBE/TZP)^40,41^ showed a much stronger interaction for **1**, while **2** had much weaker bond orders for both bridging and chelating ligands consistent with assigning the Pu complex a more lanthanide-like interaction (Tables S5–S9).

To further assess contributions to covalent metal–ligand bonding, quantum theory of atoms in molecules (QTAIM)^42^ was used to obtain the average electron density (ρ) at bond critical points, as well as delocalization indexes (δ) between metal and hydrogen atoms. Covalency in metal–ligand bonds can be influenced by changes in 1) metal–ligand orbital overlap or 2) frontier orbital energy matching (i.e., energy-degeneracy-driven covalency).^43−48^ QTAIM has been used to distinguish between these contributions. The ρ values have been used by us and others as a reflection of orbital-driven covalency,^44,49^ whereas the δ values can be used as a metric for degeneracy-driven covalency.^50−52^

QTAIM calculations indicate that U and Pu have a larger accumulation of electron density (ρ) at the M–H bond critical points compared to the lanthanides for both the chelating and bridging ligands (Figure 4; Tables S15 and S16). The increased ρ values for **1** and **2** relative to those of lanthanides are consistent with the greater orbital overlap expected due to the larger radial extension of the 6d and 5f orbitals of the actinides. Moreover, the ρ values are slightly larger with Pu than U, but they generally track along the same trend vs radius as observed with lanthanides. By contrast, the delocalization indexes (δ) suggest more favorable orbital energy matching between the bridging ligands and U in **1** compared to Pu in **2** (Figure 4; Figures S27–S29). However, we note that the decrease in coordination number from 14 in **1** to 13 in **2** also contributes to the decrease in δ. Owing to the change in coordination number, the values shown in Figure 4 represent the sum of δ per ligand instead of an average, but the individual values are presented in Figure S30. Unlike the bridging ligands, the sum of the δ values for the chelating ligands are similar for both Pu and U.

**Figure 4:**
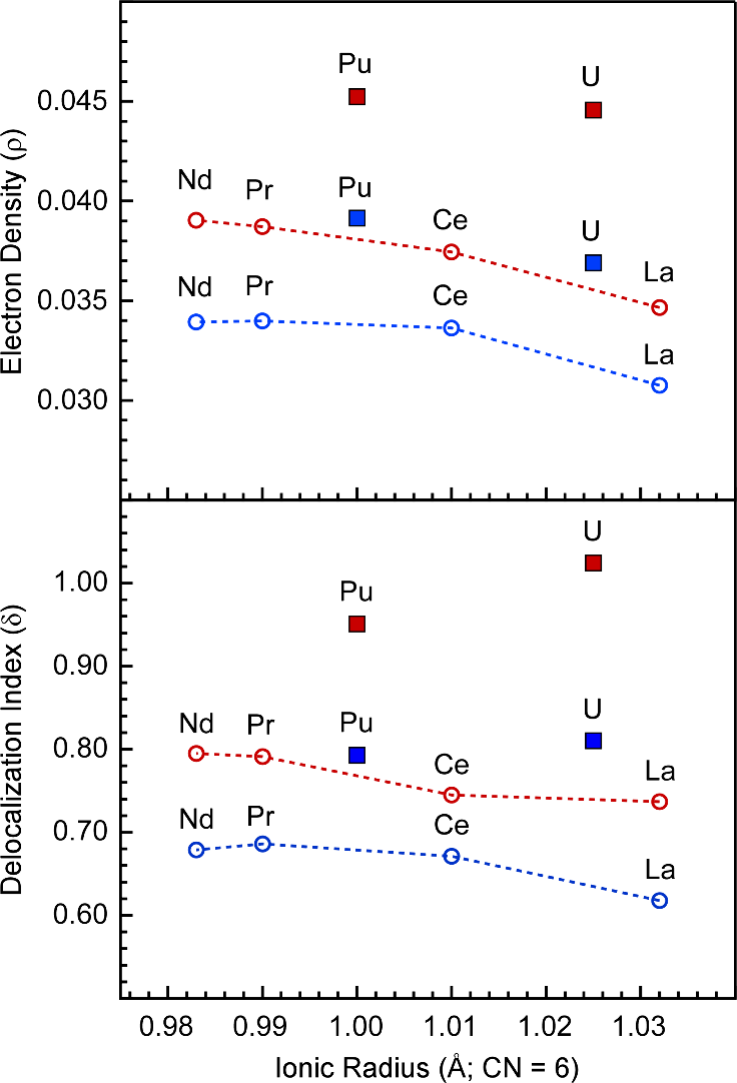
*Top* – Average QTAIM electron density (ρ) at the M–H bond critical points (PBE/TZP) for the chelating (blue) and bridging (red) ligands in the trivalent lanthanide and actinide dimers plotted as a function of ionic radius.^26^*Bottom* – Sum per ligand of the M–H delocalization indices (PBE/TZP) for the chelating (blue) and bridging (red) ligands in the trivalent lanthanide and actinide dimers. Actinides are represented by solid squares, whereas lanthanides are represented by open circles. Data points in red represent average values obtained for bridging ligands, whereas data points shown in blue represent average chelating ligands. Dashed lines between the lanthanide data points are to help guide the eye.

In summary, we have described the synthesis of Pu_2_(H_3_BP^*t*^Bu_2_BH_3_)_6_ (**2**), the first example of a structurally characterized Pu(III) borohydride complex. The single-crystal XRD data collected for **2** allowed for the first experimental comparison of borohydride complexes with different trivalent actinides. The combined structural and theoretical data suggests that covalent metal–ligand bonding with ^*t*^Bu-PDB generally decreases in the order U > Pu > lanthanides. Evidence of greater metal–ligand covalency with U over Pu was revealed in calculations of the bridging metal–ligand bonds, but the differences between U and Pu were more subtle with chelating ^*t*^Bu-PDB ligands. Efforts to expand these analyses to include Np and transuranium complexes with other borohydride ligands are currently underway.

## Data Availability

To ensure reproducibility, the input and output files associated with all calculations are available in a FigShare repository (doi.org/10.6084/m9.figshare.26997388).

## Supporting Information Available

Experimental and theoretical details; crystallographic data and spectra (PDF)
