# Supplementary material for: Structural and Theoretical Assessment of Covalency in a Pu(III) Borohydride Complex
Source: J Am Chem Soc. 2024 Sep 16;146(38):25943–8. doi: 10.1021/jacs.4c09888 (PMC11440501; doi:10.1021/jacs.4c09888)
Supplement: Supplementary file 1 — ja4c09888_si_001.pdf [file ja4c09888_si_001.pdf]

SUPPORTING INFORMATION

Structural and Theoretical Assessment of Covalency in a Pu(III) Borohydride Complex

Joshua C. Zgrabik,[a,‡] Daniel J. Lussier,[b,‡] Rina Bhowmick,[c,‡] Ngan Nguyen,[c] Peter A. Zacher III,[a] Tatyana Elkin,[b] Andrew J. Gaunt,*[b] George S. Goff,[b] Harris E. Mason,[b] Jesse Murillo,[b] Brian L. Scott,[b] Bess Vlaisavljevich,*[a,c] Scott R. Daly*[a]*

[a]University of Iowa, Department of Chemistry, E331 Chemistry Building, Iowa City, IA, 52242, United States

[b]Los Alamos National Laboratory, P.O. Box 1663, Los Alamos, NM, 87545, United States

[c]University of South Dakota, 414 E. Clark St. Vermillion, SD, 57069, United States

Table of Contents

1. Experimental Details 3

1.1. Radiological Considerations 3

1.2. Equipment, Materials, and Solvents Used for Plutonium Reactions 3

1.3. Scaled Down Synthesis of [U_2(^{t}Bu-PDB)$_6$] (**1**)........ 8

1.4. Synthesis of [Pu_2(^{t}Bu-PDB)$_6$] (**2**)........ 8

1.5. Photographs Taken During Synthetic Procedure B. 11

2. Crystallographic details........ 13

3. NMR Spectra........ 14

4. Solid-state UV-vis-NIR spectrum of 2. 20

5. Computational Details 23

5.1. Molecular Geometry of Pu Monomer and Dimer (TPSS-D3/def2-TZVP,def-TZVP) 25

5.2. Deoligimerization energies (TPSS-D3/def2-TZVP,def-TZVP) 30

5.3. Bond Order Analysis (PBE/TZP) 31

5.3. Energy Decomposition Analysis (EDA) (TPSS-D3/def2-TZVP,def-TZVP) 37

5.4. Delocalization Indices (TPSS-D3/def2-TZVP,def-TZVP) 39

5.5. Topological Analysis of the Electron Density, Quantum Theory of Atoms in Molecules (QTAIM) (TPSS-D3/def2-TZVP,def-TZVP) 41

5.6. Topological Analysis of the Electron Density, Quantum Theory of Atoms in Molecules (QTAIM) (PBE/TZP) 43

6. Supporting Information References 52

1. Experimental Details

1.1. Radiological Considerations

Caution! ^{239}Pu ($t_{½}$ = 2.411×10^5 years), and the other Pu isotopes present in sources of Pu of nominally weapons-grade isotopic composition (along with their daughters) present serious health threats due to their α-, β-, and γ-emissions. Accordingly, the primary occupational hazard of these systems is due to radiotoxic (α-particles) and heavy-metal toxicity effects. Hence, all studies that involved manipulation of these isotopes were conducted in a radiation laboratory equipped with high-efficiency particulate air (HEPA) filtered hoods and in negative-pressure gloveboxes. Additional safeguards included continuous air monitoring and use of hand-held radiation monitoring equipment. Entrance to the laboratory space was controlled with a hand and foot radiation monitoring instrumentation and a full body personal contamination monitoring station. In addition to standard laboratory personal protective equipment, fume-hood work involved multiple layers of latex gloves combined with DuPont™ Tyvek® 400 sleeves to provide overlapping coverage of the arms. Any handling of organic solvents with transuranium isotopes in a fume-hood required an additional layer of nitrile gloves over the two pairs of latex gloves.

1.2. Equipment, Materials, and Solvents Used for Plutonium Reactions

A negative-pressure, transuranium capable, helium atmosphere (glovebox fed by UHP He cylinder), MBraun Labmaster 130 glovebox was used for all work involving the synthesis of transuranium compounds. The glovebox atmosphere was maintained with a standalone Vacuum Atmosphere Genesis^{TM} oxygen and moisture removal system, and atmosphere suitability was verified using a dilute toluene solution of $[Ti(Cp)_2(\mu\text{-}Cl)]_2$ (200

mg of commercial $[Ti(Cp)_2(Cl)_2]$ reduced over an excess of Zn powder in 20 mL of toluene, and filtered) prior to any manipulations, such that the residue dried to a dark green color each time (a color change to yellow or orange indicates decomposition of the Ti test compound and atmospheric O_2/H_2O removal is required). Anhydrous THF (Sigma Aldrich), anhydrous *n*-hexane (Sigma Aldrich), and anhydrous pentane (Sigma Aldrich) were transferred onto activated 3 or 4 Å molecular sieves and degassed before use. D_6-benzene (Cambridge Isotopes) was stored over activated 4 Å molecular sieves and degassed before use – all solvents were tested with a dilute THF solution of Na_2Ph_2CO (150 mg Ph_2CO in 20 mL of THF with an excess of Na metal) such that THF required 1 drop / mL to retain purple coloration and hydrocarbon solvents required 1 drop / 2 mL. Chlorobenzene was dried over CaH_2 and distilled before use. $K(H_3BP^tBu_2BH_3)$ and $[UI_3(THF)_4]$ were prepared as described previously.[1, 2] $[PuI_3(THF)_4]$ was prepared as previously described using an aqueous Pu(IV) multi-molar hydrochloric acid aqueous stock solution as the source of plutonium.[3]

All glassware, and glass-fiber filter discs, was stored in a vacuum oven (>150 °C) for at least 24 hours prior to being brought into the glovebox, and FEP (fluorinated ethylene propylene) NMR liners were brought into the box *via* overnight or multi-hour vacuum cycles. Transuranium crystals for single-crystal X-ray diffraction were mounted in Paratone-N oil inside 0.5 mm quartz capillaries (Charles Supper). The quartz capillaries were inserted through silicone stoppers and placed inside test tubes to allow handling inside the transuranium glovebox while mounting crystals without contaminating the exterior surface of the capillary. The capillaries were then cut to appropriate size for later goniometer mounting with nail clippers. The ends of the cut capillaries were sealed with

hot capillary wax before being removed from the glovebox for coating in clear nail varnish (Hard as Nails™) to provide shatter-resilience. During the clipping and wax sealing steps, care must be taken to avoid the capillary touching any contaminated surfaces (this is achieved by the introduction of fresh petri dishes, forceps, clippers, and wax, as needed in conjunction with careful handling techniques to avoid contamination transfer).

Solid-state UV-vis-NIR measurements on single-crystals were performed on a CRAIC Technologies 2030PV PRO microspectrophotometer in transmission mode. The crystals were first transferred to petri-dish containing paratone oil inside the He-atmosphere transuranium glovebox. This dish was then brought out of the glovebox into the adjoining transuranium fume-hood. A borosilicate glass slide (containing a small pool of paratone oil and a thin square ribbon of silicone grease to act as a physical barrier to prevent transuranium crystals ‘sailing’ off the slide onto the microscope stage) was then introduced into the fume-hood on a clean Kim-towel. A minimum amount of crystals coated in oil were then transferred from the petri-dish using a spatula into the pool of paratone oil on the quartz slide being careful not to touch anywhere else on the slide other than the pool of paratone. The edges and surfaces of the slide, outside of the grease barrier, were then smeared and the smear counted first on a hand-held α-particle detector and then on a more sensitive 3030E Ludlum smear counter to ensure the parts of the slide that would be handled and contact the microscope stage were free of contamination before transport to the instrument in a new petri-dish. Following data acquisition, the microscope stage was surveyed and smeared/counted to check for contamination.

For NMR spectroscopy, a C_6D_6 solution of the compound was loaded into a fresh FEP NMR liner (using a plastic pipette tip inserted into the top of the liner to protect the

exterior of the liner at the top from contamination during loading and then removal of the glass transfer pipette) that was protected from surface contamination with Parafilm while inside a transuranium glovebox. The liner was sealed with two PTFE plugs (the upper most one partially protected by masking tape) after first tapping the liner to ensure all liquid was below the level that the PTFE plugs would be inserted. The liner was then brought out of the glovebox, the parafilm removed, and the exterior of the liner verified to be free of surface contamination (first by direct α-probe survey then by taking a smear and counting on the 3030E Ludlum instrument). In this case, as we often find with plutonium samples of primarily ^{239}Pu isotopic composition, it was necessary to use scissors to cut off a few millimeters from the top of the liner to remove contamination (this is in contrast to most of ^{237}Np NMR samples where often the top of the liner is found to be free of contamination after unwrapping the parafilm – thus sample preparation and release requirements to an instrument may vary depending upon the transuranium isotope and specific sample – not all transuranium samples can follow a standard protocol and must be dealt with on a case by case basis in real time). The liner was then loaded into a J. Young tap appended 5 mm NMR tube protected inside a plastic bag taped to the tube and the sealing value was wrapped in masking tape and placed inside a covered petri-dish. Both of these items were placed in the antechamber of the transuranium glovebox and the headspace of the tube was then evacuated and refilled with He three times to provide an inert atmosphere headspace above the sample using standard antechamber cycles. The tube was then sealed with the valve inside the glovebox before being brought back out into the transuranium fumehood. Here, the wrapping materials were removed from the NMR tube and the tube was surveyed and smeared as above to

ensure no contamination. Since the outermost containment layer (the J-Young valved NMR tube) had been physically inside of the transuranium glovebox (albeit protected by wrapping), parafilm was wrapped around where the valve connects to the tube because it is difficult to effectively smear up in the screw-thread and the tube was always handled wearing gloves and kept sealed in a plastic bag when not inside the spectrometer.

NMR spectroscopic data were collected on the following nuclei—^{1}H, $^{31}P\{^{1}H\}$, and $^{11}B\{^{1}H\}$—with a 400 MHz Bruker AVANCE III NanoBay spectrometer using a Bruker BBO probe configured for 5 mm (o.d.) tubes. ^{1}H spectra were collected numerous times to ensure that the sample did not decompose during the longer $^{31}P\{^{1}H\}$ and $^{11}B\{^{1}H\}$ spectra acquisition times. The $^{11}B\{^{1}H\}$ spectra were collected at a resonance frequency of 128.38 MHz using a TRIP excitation scheme to reduce the appearance of background signals from the probe.[4] This sequence only suppressed the signal outside of the NMR coil, so the signal from the borosilicate glass J-Young tube remains in the final spectrum. To ensure no degradation of sample occurred during data collection, the $^{11}B\{^{1}H\}$ data were collected as a series of 9 spectra with 2000 acquisitions each, then summed to produce the final spectrum. The $^{31}P\{^{1}H\}$ spectra were collected at a resonance frequency of 161.98 MHz. To ensure no sample degradation occurred during data collection, the $^{31}P\{^{1}H\}$ data were collected as a series of 2 spectra with 15,000 acquisitions each, then summed to produce the final spectrum. Chemical shifts for ^{1}H resonances are reported in δ units (i.e. ppm) relative to residual solvent (C_6D_6) peaks.[5] The $^{31}P\{^{1}H\}$ and $^{11}B\{^{1}H\}$ signals were internally referenced to these residual solvent peaks via the following equation, which is the IUPAC recommended convention.

$$\Delta\,(Hz) = \frac{SR^{1H}}{SF^{1H}} \cdot SF^{NUC}$$

Where SR^{1H} is the spectrum reference frequency (in Hz) of a reference ^{1}H NMR spectrum collected with TMS set to 0 ppm collected under the same experimental conditions, SF^{1H} is the spectrometer frequency (in MHz) for the ^{1}H nucleus, and SF^{NUC} is the spectrometer frequency (in MHz) of the nucleus in question. The answer is given in Hz and converted to ppm.

*1.3. Scaled Down Synthesis of [U_2(^{t}Bu-PDB)$_6$] (**1**).*

In a 1.5-dram vial, UI_3(THF)$_4$ (9 mg, 0.01 mmol) and K(^{t}Bu-PDB) (6.3 mg, 0.03 mmol) were dissolved in dry chlorobenzene (1 mL). The solution was stirred overnight, filtered through a pad of celite, and evaporated to dryness. The solid was dissolved in the minimum amount of pentane (~1 mL) and placed in a fridge at -30°C to yield red crystals. Yields: 3 mg (run 1, 40%), 5.5 mg (run 2, 79%). Additional information: U_2(^{t}Bu-PDB)$_6$ is soluble in pentane but requires some stirring to fully dissolve the sample for crystallization. The crystals formed were mostly microcrystalline but there were several larger crystals of XRD quality when checked under the microscope in oil. ^{1}H and ^{11}B NMR data collected on the crystals matched those reported previously.[6]

*1.4. Synthesis of [Pu_2(^{t}Bu-PDB)$_6$] (**2**).*

The scaled down uranium synthetic procedure was used as a guide for the plutonium chemistry out of both radiological and conservation/efficient use of limited isotope resources considerations. The plutonium reaction was performed twice – the first with the sole goal to obtain a publishable structure derived from single-crystal X-ray

diffraction (thus diverting all of the small amounts of plutonium compound isolated into paratone oil for X-ray studies) and the second with the goal of acquiring supporting spectroscopic data (syntheses A and B, respectively).

Synthesis A: Three equivalents of solid K[^{t}Bu-PDB] (0.0104 g, 0.050 mmol) in a 4 mL glass vial were added to a 20 mL glass vial charged with [$PuI_3(THF)_4$] (0.0151 g, 0.017 mmol) and a Teflon-coated magnetic stir bar. The 4 mL vial was rinsed with chlorobenzene (1 mL) and the rinse solution added to the 20 mL vial. The reaction mixture was stirred at ambient temperature resulting in a cloudy blue mixture after a few minutes. Stirring at ambient temperature was continued overnight. The cloudy blue mixture was filtered through a glass fiber circle packed into a glass pipette resulting in a light blue filtrate. The solid that remained in the filter (presumably KI) was rinsed with ~0.6 mL chlorobenzene into the filtrate; the solid on the filter remained stained with a blue color. Volatiles were removed in vacuo from the filtrate. The pale blue solid that resulted was extracted by stirring with pentane (~5 mL) and again filtered through a glass fiber circle packed into a glass pipette resulting in a light blue filtrate. The filtrate was stored at –35 °C in the glovebox freezer resulting on deposition of pale blue needles overnight. Several of these needles were mounted in capillaries as described above and subjected to single-crystal X-ray diffraction studies – they were not of sufficient quality for full structural determination but diffraction data was able to return the same unit cell as [U_2(^{t}Bu-PDB)$_6$].The supernatant was pipetted away from the remainder of the needle crystals that were not mounted for X-ray diffraction studies. The needle crystalline material was then dissolved in ~1.25 mL n-hexane/0.2 mL Et_2O with stirring and the solution filtered through a glass fiber circle packed into a glass pipette, and the vial/filter rinsed with ~0.3 mL n-

hexane, resulting in a pale blue filtrate. After storage at –35 °C in the glovebox freezer for several days, pale blue plates deposited which were determined to be [Pu_2(^{t}Bu-PDB)$_6$] by single-crystal X-ray diffraction.

Synthesis B: Three equivalents of solid K[^{t}Bu-PDB] (0.0188 g, 0.090 mmol) in a 4 mL glass vial were added to a 20 mL glass vial charged with [PuI_3(THF)$_4$] (0.0271 g, 0.030 mmol) and a Teflon-coated magnetic stir bar. The 4 mL vial was rinsed with chlorobenzene (1 mL) and the rinse solution added to the 20 mL vial. The reaction mixture was stirred at ambient temperature resulting in a cloudy blue mixture after a few minutes. Stirring at ambient temperature was continued overnight. The cloudy blue mixture was filtered through a glass fiber circle packed into a glass pipette resulting in a cloudy light blue filtrate indicating that some solid has broken through the filter. The solid that remained in the filter (presumably KI) was rinsed with ~0.2 mL chlorobenzene into the filtrate; the solid on the filter remained stained with a blue color. Volatiles were removed in vacuo from the filtrate. The blue/white solid was extracted into ~3.5 mL pentane with stirring at ambient temperature for 5 minutes. The cloudy solution was filtered through a glass fiber circle packed into a glass pipette, then the vial and filter rinsed with 1 mL pentane, resulting in a clear pale blue filtrate. Due to the time taken to filter some pentane evaporated during filtration, meaning that the final filtrate volume was ~2 mL. The filtrate was stored at –35 °C in the glovebox freezer overnight resulting in deposition of a pale blue crystalline material. A few of the crystals were pipetted into a separate vial and used to acquire solid-state UV-vis-NIR data. The supernatant was removed from the remainder of the crystalline material, which was dried in vacuo to afford a microcrystalline pale blue powder of [Pu_2(^{t}Bu-PDB)$_6$] (0.0019 g, 8.5% yield based on Pu), which was dissolved in

~0.5 mL C_6D_6 for NMR spectroscopic measurements. Volatiles were removed in vacuo from the supernatant to afford a pale blue oily residue (0.0047 g). Attempts to isolation additional crystalline material or powder by working up this residue were unsuccessful.

1.5. Photographs Taken During Synthetic Procedure B.

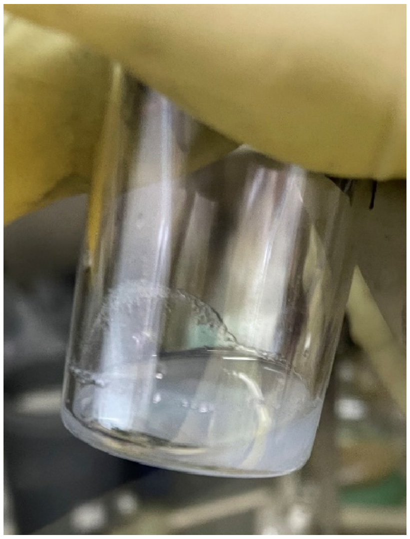

Figure S1. Pu reaction mixture after stirring overnight at ambient temperature.

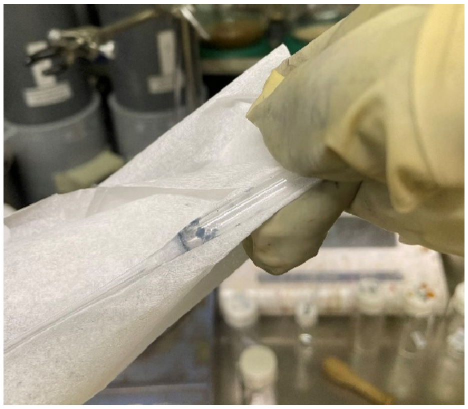

Figure S2. ‘Stained’ solid on filter (presumably KI) after rinsing with chlorobenzene.

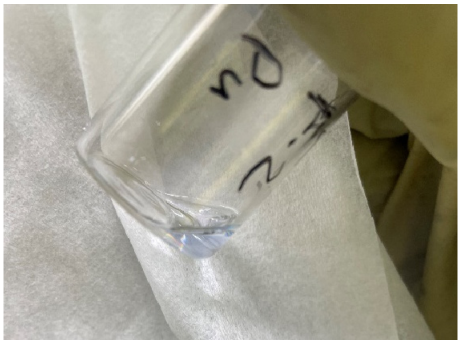

Figure S3. 1.9 mg of [Pu_2(^{t}Bu-PDB)$_6$] dissolved in C_6D_6.

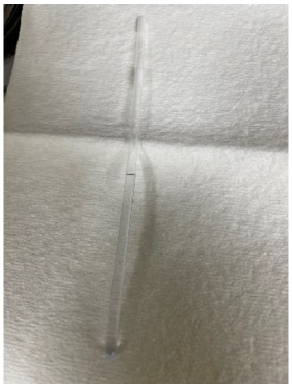

Figure S4. C_6D_6 solution of [Pu_2(^{t}Bu-PDB)$_6$] loaded into an FEP NMR tube liner with two PTFE plugs.

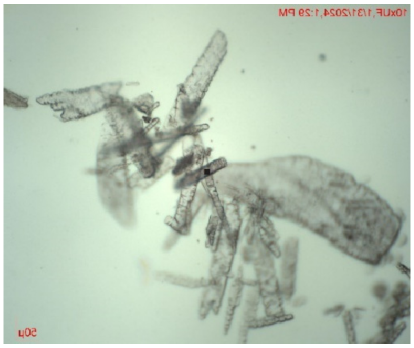

Figure S5. Crystals of [Pu_2(^{t}Bu-PDB)$_6$] used to acquire solid-state UV-vis-NIR data. The black square indicates where the area of the crystal that was probed by the CRAIC spectrophotometer.

2. Crystallographic details

A crystal of **2** was coated with paratone-N oil and placed in a capillary that was then sealed at both ends with wax. The capillary was then coated with acrylic to achieve triple containment. The data were collected at 100 K on a Bruker D8 diffractometer with a Photon II detector and a MoKα X-ray microsource. A hemisphere of data was collected using ω scans, with 0.5° frame widths. Data collection and initial indexing and cell refinement were handled using APEX 4[7] software. The data were corrected for absorption using redundant reflections and the SADABS[8] program. The structure was solved using Direct methods and difference Fourier techniques. All hydrogen atom positions were idealized, and rode on the atom they were attached to, with B-H distances set to 1.20 Å. The final refinement included anisotropic temperature factors on all non-hydrogen atoms. Two disordered hexane solvent molecules per dimer were treated using PLATON/SQUEEZE.[9, 10] Structure solution, refinement, and creation of publication materials were performed using SHELXTL.[11] The graphic provided in Figure 2 was generated using Mercury 2023.2.0.[12]

Calculation of error bars on average bridging M-B distances. The error bars (σ_{total}) for the average bridging M-B distances were calculated using the standard deviation of the averaged M-B distances (σ_{avg}) and the error (esd) values for the two independent M-B distances (σ_{esd}), as shown in Equation S1.

$$\sigma_{total} = \sqrt{\sigma_{avg}^2 + \sigma_{esd}^2} \qquad \text{(Eq. S1)}$$

The latter σ_{esd} values were calculated for each complex using the weighted standard deviation equation shown in Equation S2.[13]

$$\sigma_{esd} = \frac{1}{\sqrt{\Sigma_{1\to n} w_n}} \qquad w_n = \frac{1}{\sigma_n^2} \qquad \text{(Eq. S2)}$$

Using the bridging Pu-B distances of 2.678(5) and 2.675(6) Å yields 0.004 Å, as shown in the sample calculation below.

$$\sigma_{total} = \sqrt{(0.002\mathbf{1}\ \text{Å})^2 + (0.003\mathbf{8}\ \text{Å})^2} = 0.004\ \text{Å}$$

3. NMR Spectra

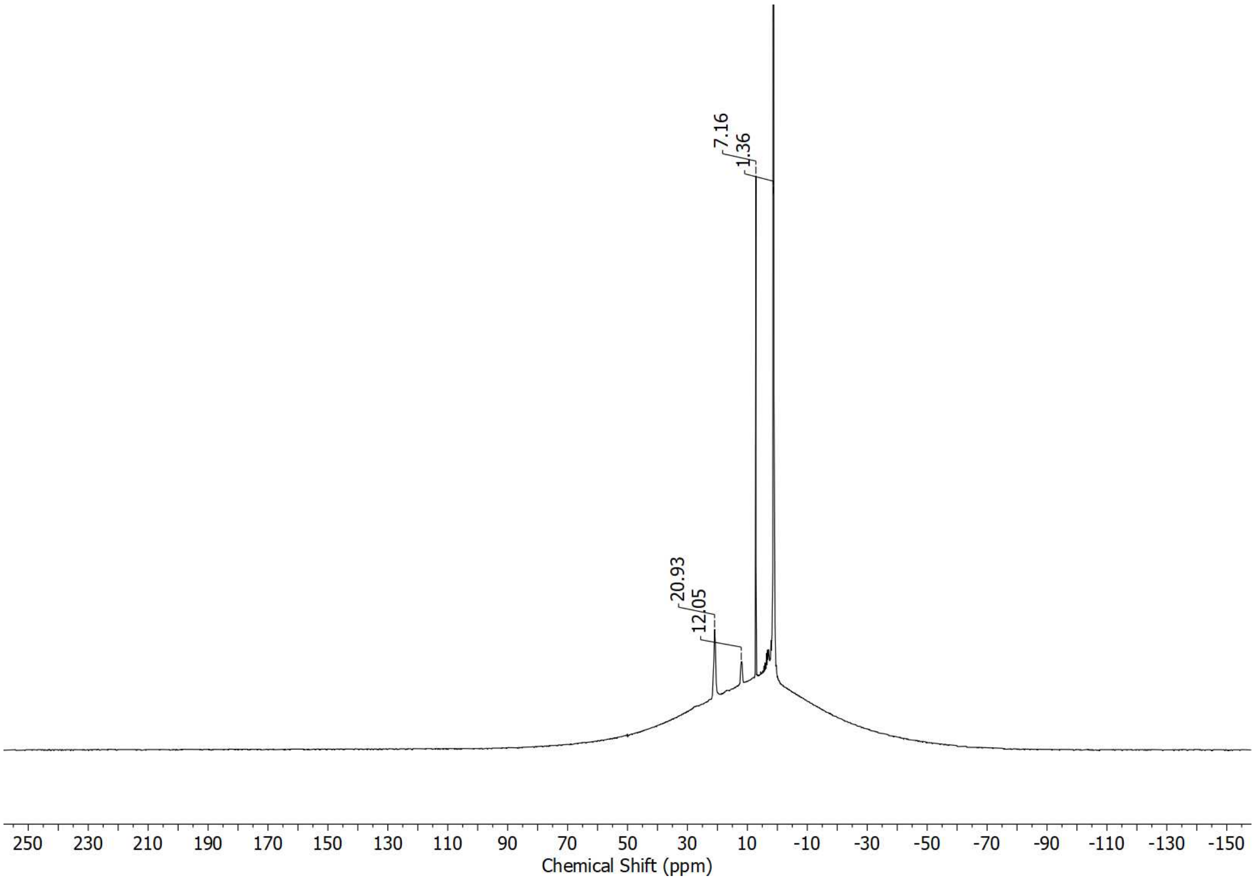

Figure S6. ^{1}H NMR spectrum of [Pu_2(^{t}Bu-PDB)$_6$] in C_6D_6 (400 MHz) with a 10 Hz exponential line broadening function applied to improve visibility of broad signals.

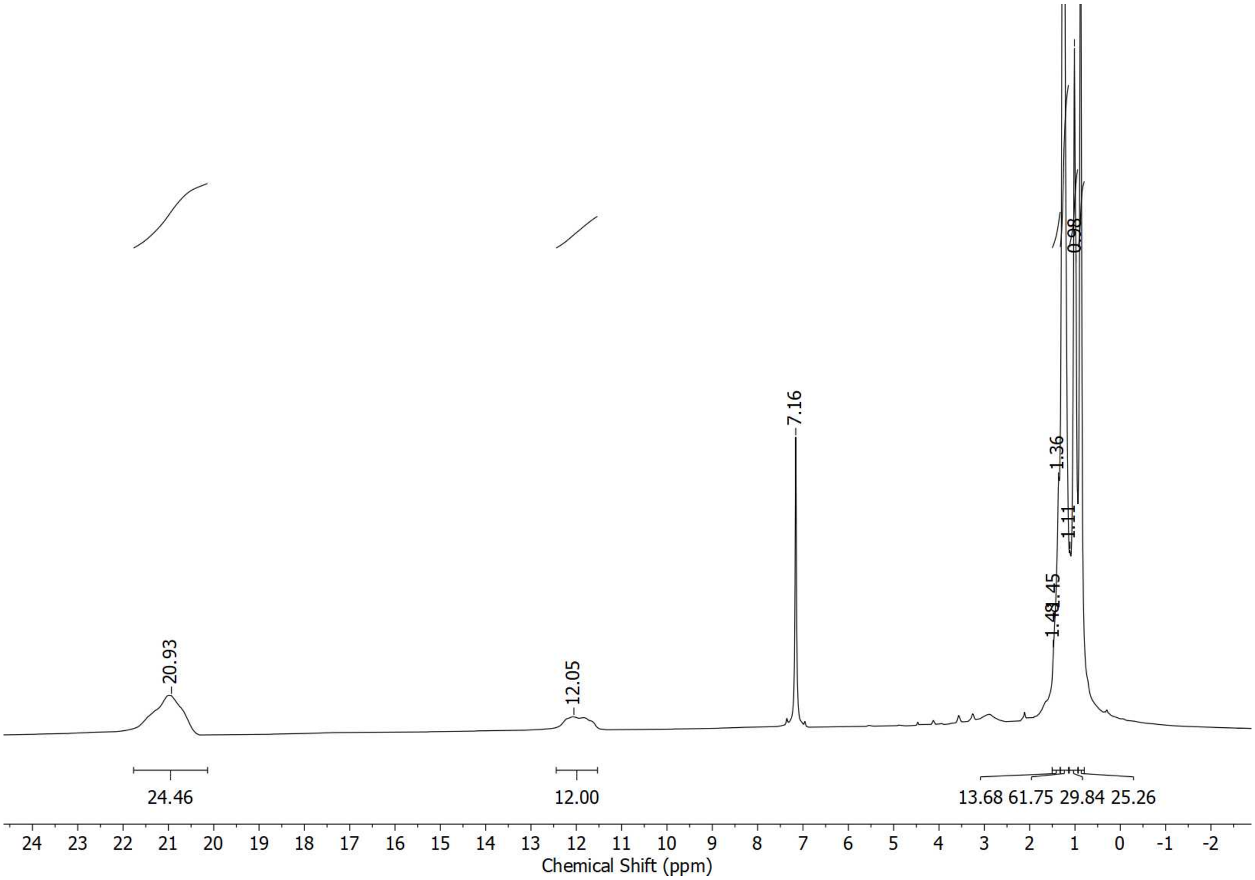

Figure S7. ^1H NMR spectrum of $[Pu_2(^tBu\text{-}PDB)_6]$ in C_6D_6 (400 MHz) with a 10 Hz exponential line broadening function applied to improve visibility of broad signals. Peaks at δ = 20.93 and 12.05 can be attributed to BH_3 chelating (24 H) and bridging (12 H), respectively. There are no obvious peaks that can be attributed to BH_3 groups in monomeric $Pu(^tBu\text{-}PDB)_3$.

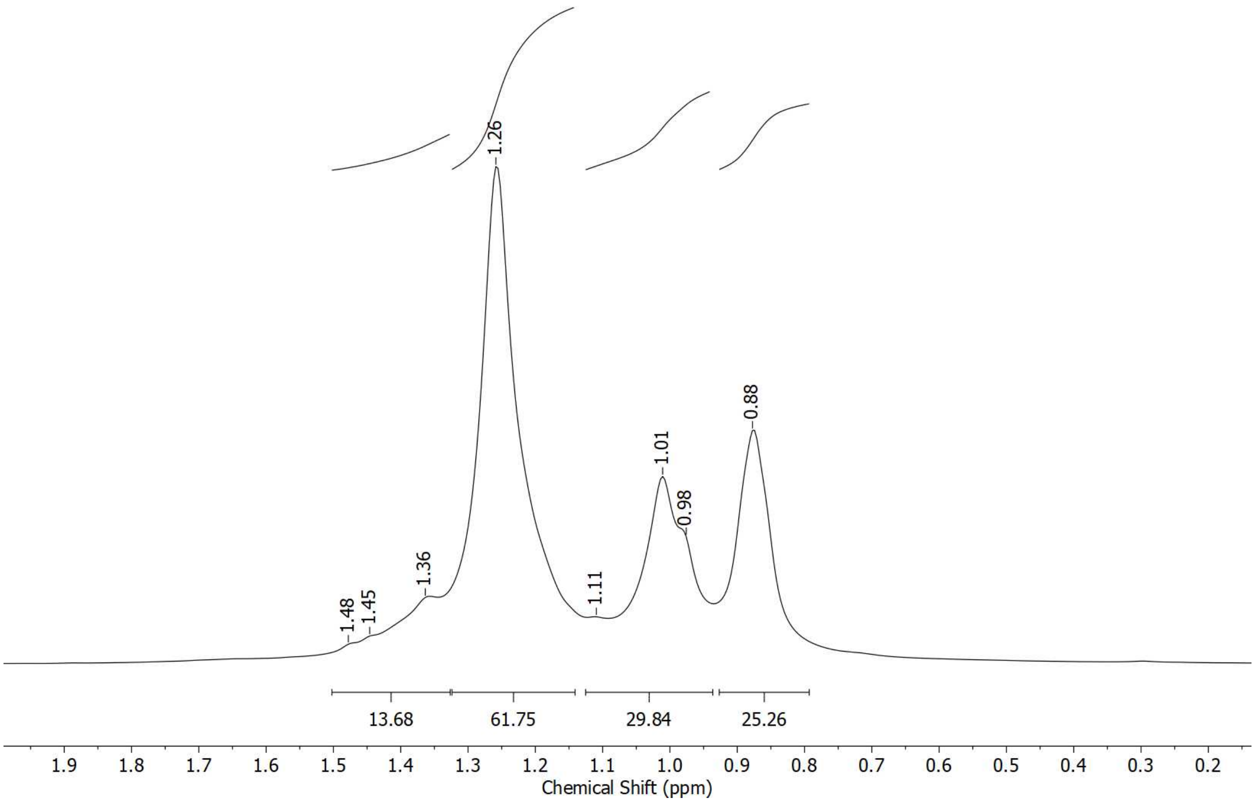

Figure S8. ^{1}H NMR spectrum of [Pu_2(^{t}Bu-PDB)$_6$] in C_6D_6 (400 MHz) in the 0.2 to 1.9 ppm region; a 10 Hz exponential line broadening function was applied to improve visibility of broad signals. Three main peaks centered around δ = 1.26, 1.01 and 0.98 ppm can be attributed to ^{t}Bu groups in ^{t}Bu-PDB. Peak assignments for [Pu_2(^{t}Bu-PDB)$_6$] (should have two ^{t}Bu peaks with 36 and 64 H for bridging and chelating ^{t}Bu-PDB, respectively) are made difficult by the presence of monomeric Pu(^{t}Bu-PDB)$_3$, small amounts of ^{t}Bu-PDB hydrolysis (observed more clearly in Figures S9 – S10), and trace amounts of organic solvents, such as n-pentane (δ = 0.87 and 1.23).[5]

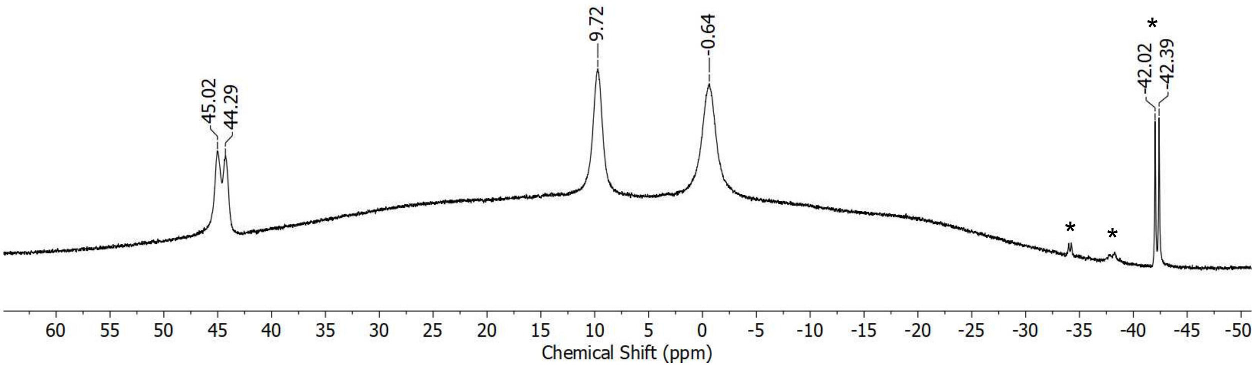

Figure S9. $^{11}B\{^{1}H\}$ NMR spectrum of $[Pu_2(^{t}Bu\text{-}PDB)_6]$ in C_6D_6. There is a broad feature between + 60 and −40 ppm due to signals from the borosilicate glass. The asterisks indicate resonances assigned to boron containing impurities from hydrolyzed ^{t}Bu-PDB.

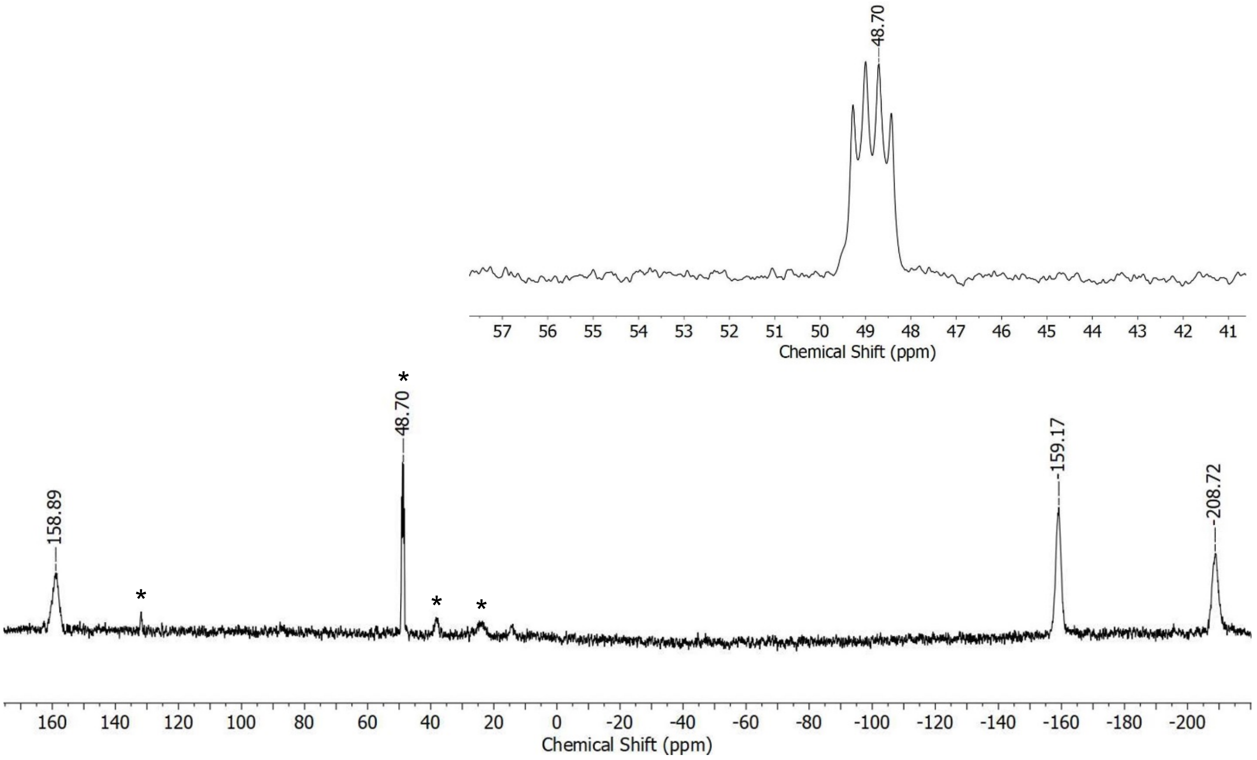

Figure S10. $^{31}P\{^{1}H\}$ NMR spectrum of $[Pu_2(^{t}Bu\text{-}PDB)_6]$ in C_6D_6. A 10 Hz exponential line broadening was applied to improve visibility of broad signals. The asterisks indicate resonances assigned to phosphorus containing impurities from hydrolyzed ^{t}Bu-PDB.

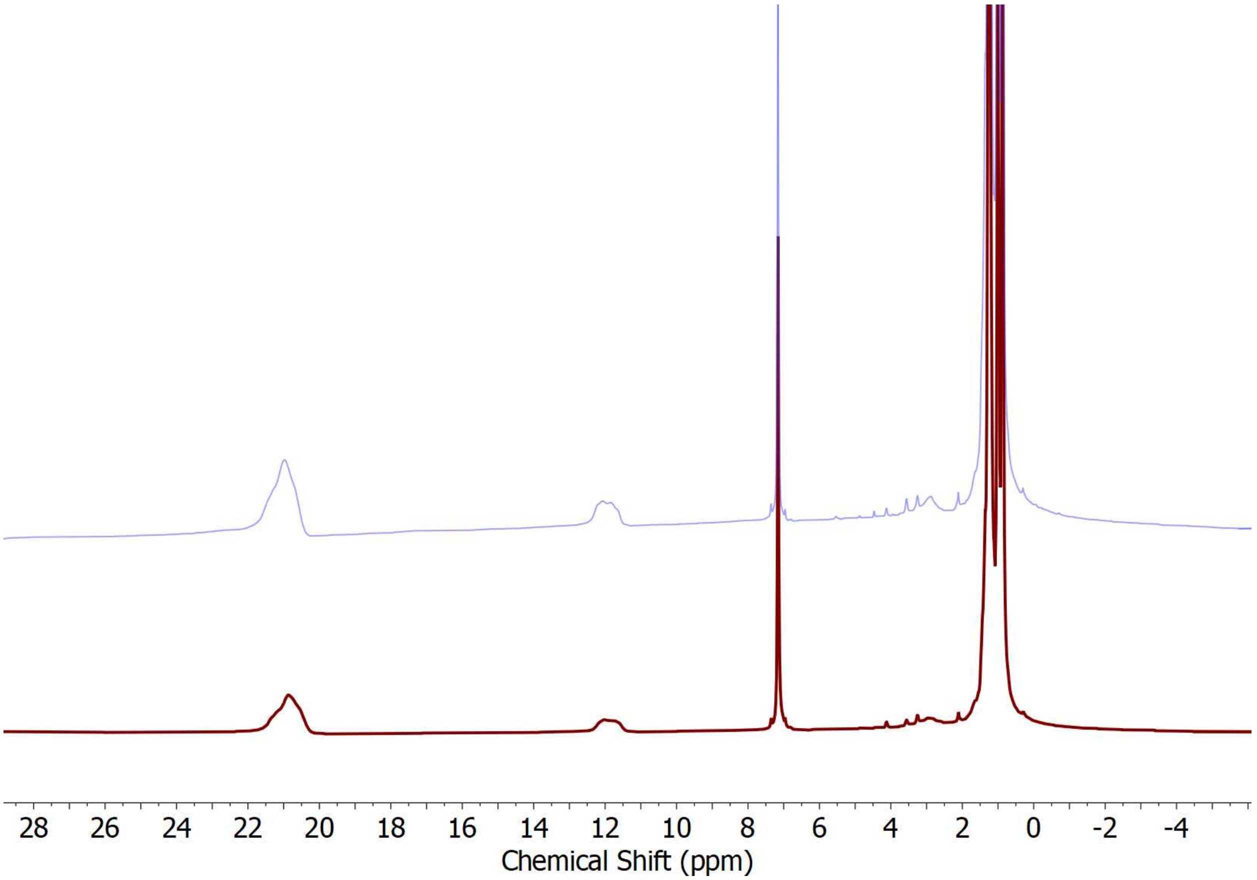

Figure S11. Stacked ^{1}H NMR spectra of $[Pu_2(^{t}Bu\text{-}PDB)_6]$ in C_6D_6 (400 MHz) taken at the beginning of the NMR experimental run (light blue curve) and after completion of the long scan $^{31}P\{^{1}H\}$ and $^{11}B\{^{1}H\}$ measurements (maroon curve), illustrating that the sample did not decompose; a 10 Hz exponential line broadening function was applied to improve visibility of broad signals.

4. Solid-state UV-vis-NIR spectrum of 2.

Solid-state UV-vis-NIR spectra were recorded on single-crystals of **2** (see Figures S12 and S13 for a representative spectrum of one crystal). 5f-5f transitions were observed that are consistent with assignment of a compound containing the Pu(III) oxidation state based on literature comparison. Bands in the ~400-700 nm region closely resemble several spectra reported for Pu(III) molecules, while bands in the ~750-1400 nm region are broader but still generally consistent with other reported Pu(III) molecules.[3, 14-18] The solution-phase UV-vis-NIR spectra was not recorded due to the combination of small reaction scale/yield and the fact that NMR data suggested that multiple species were present in solution.

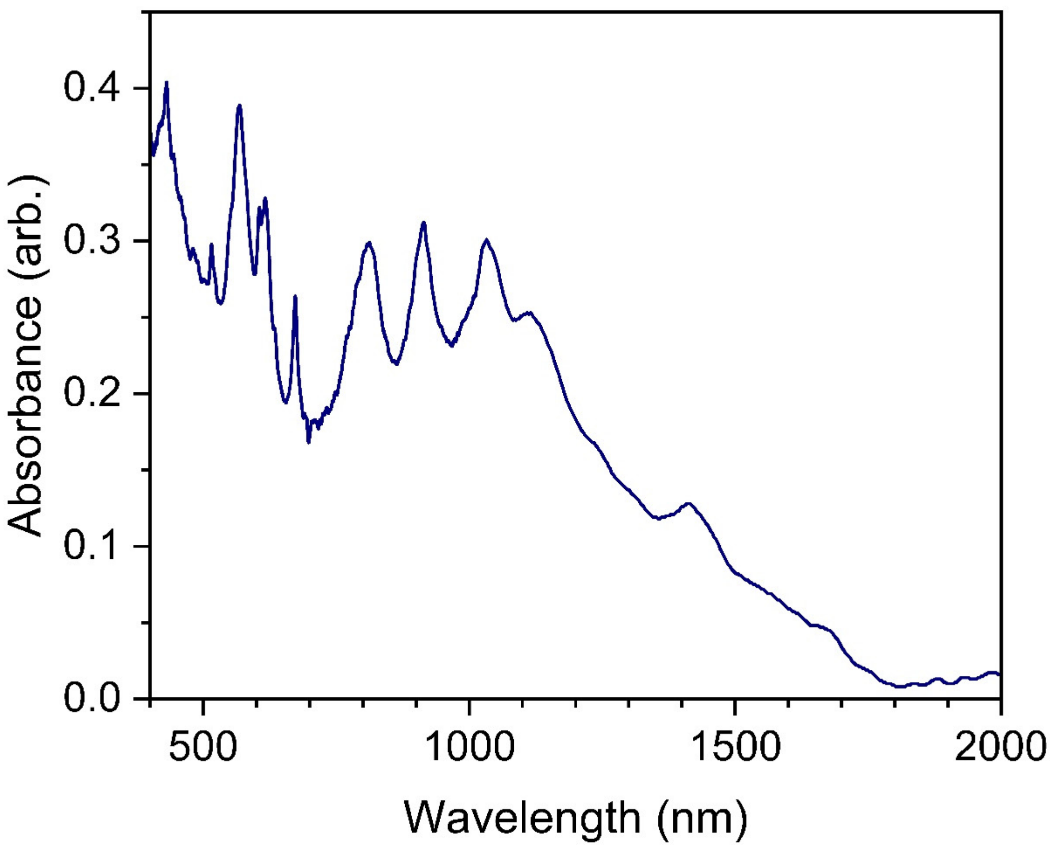

Figure S12. Solid-state UV-Vis-NIR spectrum of [Pu_2(^{t}Bu-PDB)$_6$] with wavelength represented on the x-axis.

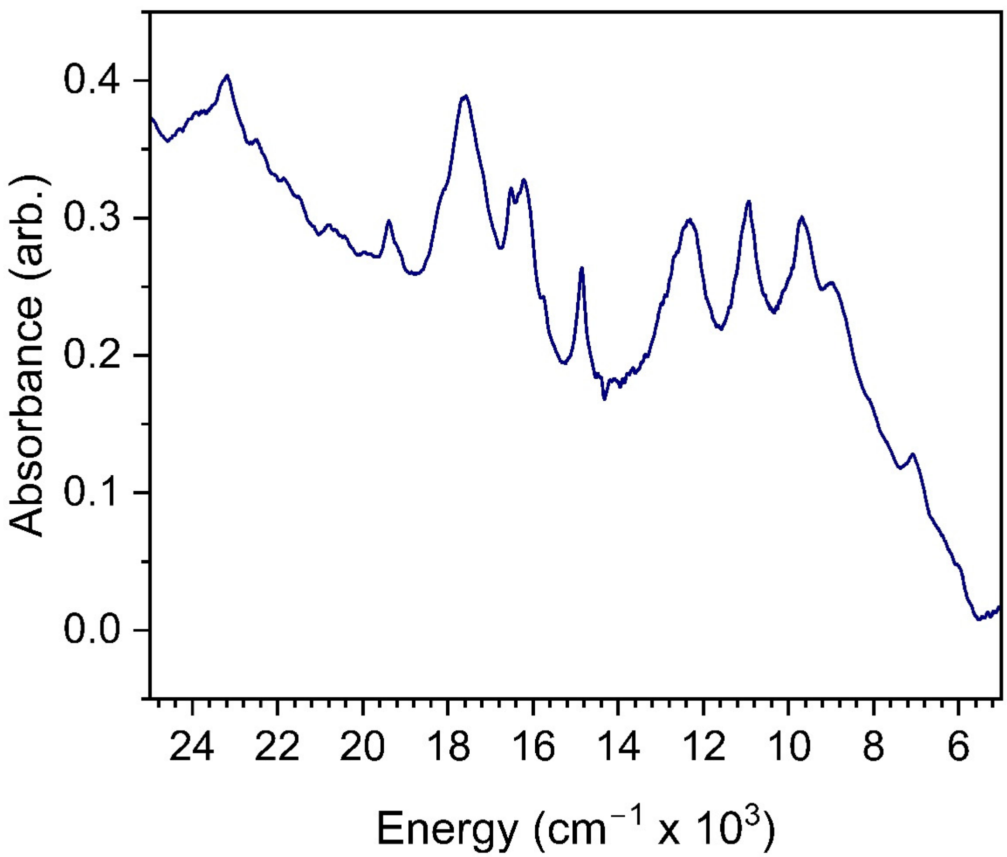

Figure S13. Solid-state UV-Vis-NIR spectrum of [Pu_2(^{t}Bu-PDB)$_6$] with wavenumber represented on the x-axis.

5. Computational Details

Starting structures for the Pu monomer and dimer were taken from the previously optimized structures for U in Ref 19 employing the same level of theory to allow direct comparison between the two studies. First, gas phase geometry optimizations were performed with density functional theory (DFT). The TPSS meta GGA functional was employed with Grimme's D3 correction and the original damping function, denoted TPSS-D3.[20, 21] The resolution of the identity (RI) approximation was used for integral evaluation.[22] The def2-TZVP basis set was used for all atoms with the exception of Pu where the def-TZVP basis set was used.[23-29] The corresponding effective core potentials (ECPs) are employed to recover scalar relativistic effects. The SCF energy was converged to 10^{-7} a.u., and the Cartesian gradient was converged to 10^{-4} a.u. All structures were confirmed as minima by harmonic vibrational analysis. Free energies are reported using the standard harmonic oscillator and rigid rotor approximations. Subsequent single point calculations including the conductor-like screening model (COSMO)[30] were performed on the gas phase geometries to account for solvation using a dielectric constant of 2.274 for benzene. These DFT calculations were performed as implemented in the Turbomole program package.[31] The enthalpy and free energy associated with the dimer deoligomerization were computed including a quasiharmonic correction suggested by Cramer and Truhlar.[32] Specifically, all normal modes less than 100 cm^{-1} were replaced with 100 cm^{-1}. Additionally, the free energies were corrected using the single point energies in benzene, computed at 298.15K, and assuming a concentration of 1 M for all reactants and products. A topological analysis of the electron density was performed on the with the Bader's Quantum Theory of Atoms in Molecules (QTAIM)[33] and delocalization indices are reported.

To further assess the bonding between plutonium and the borohydride groups, the complexes were further characterized by performing single point calculations and an energy decomposition analysis (EDA) as implemented in the Amsterdam Density Functional program[34] using the PBE[35, 36] functional. The TZP basis set was used on all the atoms and relativistic corrections were taken into account using the scalar relativistic zero-order regular approximation (ZORA).[37] No core electrons were frozen. The fragments in EDA were defined as one PDB ligand as the anionic fragment and the remainder of the molecule as the cationic fragment. For the monomer, only one EDA calculation is performed since all PDB ligands are chelating; however, for the dimer, two EDA calculations are performed since the bridging and chelating ligands are in different environments. The EDA calculations were also performed for Nd, Pr, Ce, La, and U since this analysis was not included in the prior study. On the Pu monomer and dimer, Nalewajski-Mrozek bond orders (version 3) are also computed in ADF using the same level of theory. Calculations were also performed for the La, Ce, Pr, Nd, and U dimers on the previously reported optimized geometries.[19] QTAIM analysis, including delocalization indices, were also performed as implemented in ADF. The prior computations using MultiWFN[38] resulted in bond critical points (BCPs) localized between the M and B atoms; however, in ADF the BCPs corresponds to BCPs connected to M and H atoms. We include both analysis for completeness, although the trends obtained remain the same in both cases.

5.1. Molecular Geometry of Pu Monomer and Dimer (TPSS-D3/def2-TZVP,def-TZVP)

The DFT optimized structure for **2** generally reproduces the experimental structure. The chelating distances (κ^2-BH_3), for example, are in good agreement with experiment, ranging from 2.788 – 2.868 Å (Table S1). The calculated bridging ligands differ slightly in that they reveal asymmetry with one κ^2-BH_3 (Pu-B 2.773 Å) and one κ^3-BH_3 (Pu-B 2.599 Å) to give an overall coordination number of 13 for each Pu. Despite this difference, the average Pu-B bridging distance is 2.686 Å (a 0.009 Å deviation from the average experimental value).

Table S1. Selected geometric parameters of the Pu dimer and monomer from the optimized geometry using RI-TPSS-D3/def2-TZVP, def-TZVP for Pu. Distances in Å.

	**Monomer**		**Dimer Pu 1**		**Dimer Pu 2**	
**Ligand**	**Pu-B**	**Pu-H**	**Pu-B**	**Pu-H**	**Pu-B**	**Pu-H**
1	2.795	2.286	2.813	2.459	2.795	2.292
		2.447		2.308		2.463
		3.874		3.888		3.886
	2.836	2.418	2.868	2.500	2.788	2.339
		2.414		2.395		2.399
		3.931		3.959		3.884
2	2.838	2.418	2.788	2.339	2.813	2.459
		2.415		2.400		2.308
		3.932		3.885		3.888
	2.795	2.285	2.794	2.463	2.868	2.395
		2.447		2.295		2.501
		3.873		3.886		3.959
3	2.712	2.301	2.599 [a]	2.321	2.773 [a]	2.412
		2.409		2.438		2.273

		3.858		2.593		3.378
	2.713	2.301	2.773 [a]	2.273	2.599 [a]	2.320
		2.410		2.412		2.438
		3.858		3.376		2.594

[a]Corresponds to a bridging ligand.

Table S2. Average geometric parameters of the Pu dimer and monomer from the optimized geometry using RI-TPSS-D3/def2-TZVP, def-TZVP for Pu. Standard deviation is reported. Distances in Å and angles in degrees

Parameter	Ligand Type	Monomer	Dimer
Pu-B	chelating	2.782 ± 0.057	2.816 ± 0.034
B-Pu-B	chelating	107.8 ± 3.6	106.0 ± 0.3
Pu-B	bridging		2.686 ± 0.100
B-Pu-B	bridging		118.2 ± 0.0

Table S3. Average geometric parameters of the Pu dimer and monomer from the optimized geometry using RI-TPSS-D3/def2-TZVP, def-TZVP for Pu. Standard deviation is reported. Distances in Å. Note that H_a refers to the longest distance with H_b, and H_c following in decreasing order.

Parameter	Ligand Type	Monomer	Dimer
Pu-H_a	chelating[a]	3.888 ± 0.035	3.904 ± 0.034
Pu-H_b	chelating	2.427 ± 0.016	2.455 ± 0.038
Pu-H_c	chelating	2.332 ± 0.060	2.334 ± 0.042
Pu-H_a	bridging[a]		3.377 ±0.001
Pu-H_b	bridging		2.379 ± 0.065

[a]This distance corresponds to Pu-H distances in which the H atom is not coordinated; therefore, it was averaged separately from the coordinated H atoms.

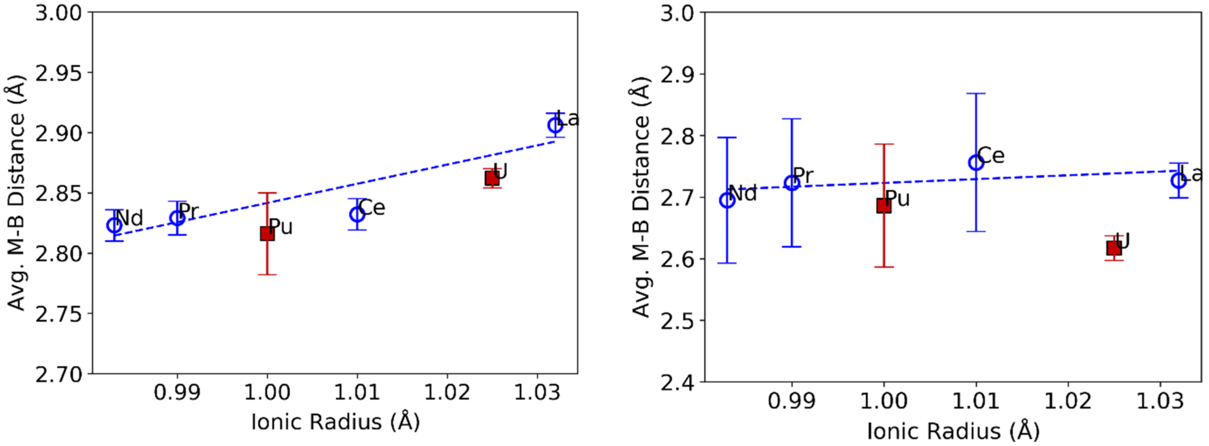

Figure S14. Average M-B bond distances for the chelating (left) and bridging (right) ligands. Data for other metals from Ref [19]. Standard deviations are shown as error bars. Distances in Å. Note that the large error bars on the bridging M-B distances appear for the complexes where both κ^2 and κ^3 bridging groups are present resulting in asymmetry.

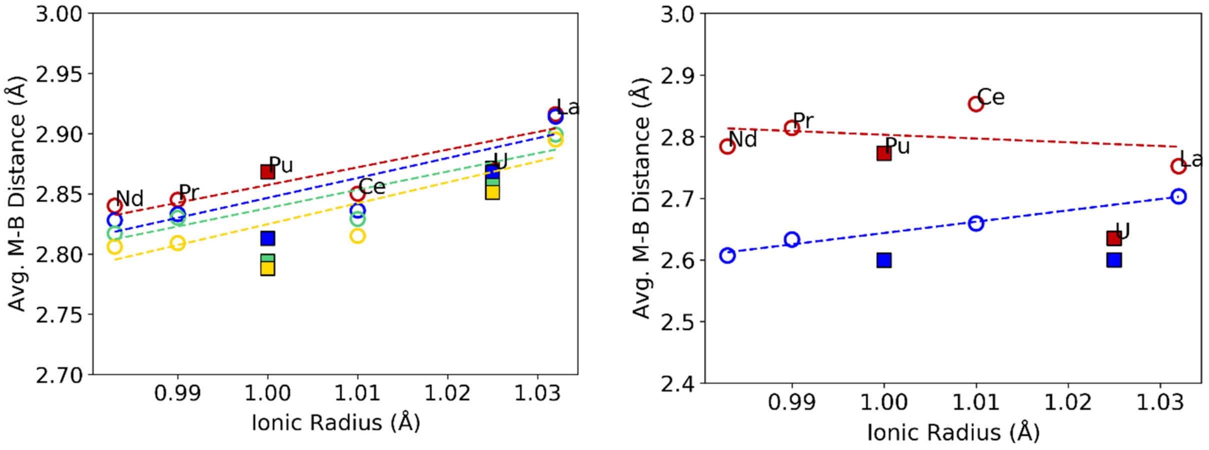

Figure S15. M-B bond distances for the chelating (left) and bridging (right) ligands equivalent bonds on the two metal centers are averaged). Data for other metals from Ref [19]. Distances in Å. Note that in the plot on the right, the two bridging U-H_3B and La-H_3B groups are both κ^3. In Pu and the smaller lanthanides (Ce – Nd), the shorter M-B distance in blue is κ^3 and the longer M-B distance in red is κ^2.

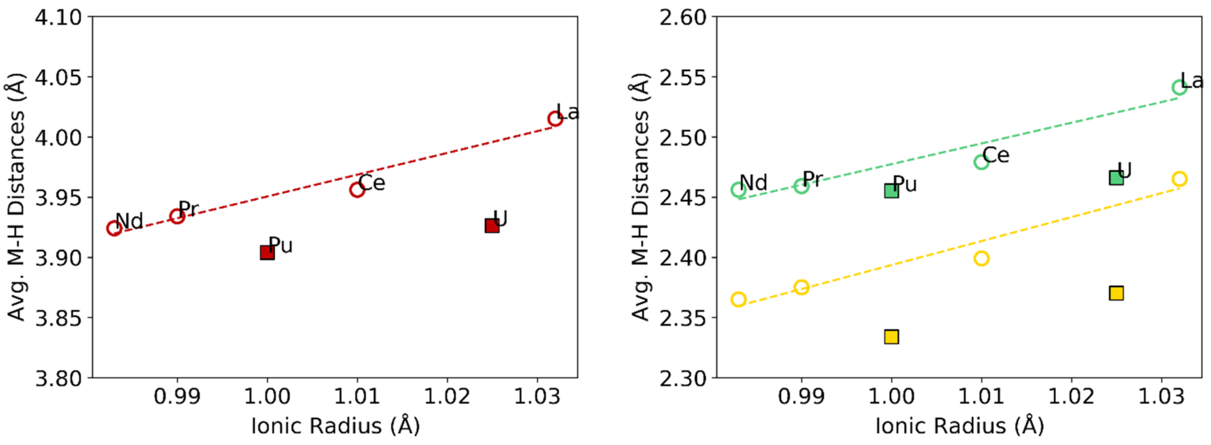

Figure S16. M-H bond distances for the chelating ligand. The distance with the hydrogen atom that is not coordinated to the metal is included on the left, while the distances with the two coordinated hydrogen atoms are plotted on the right. Equivalent bonds on the two metal centers are averaged together. Data for other metals from Ref [19]. Distances in Å.

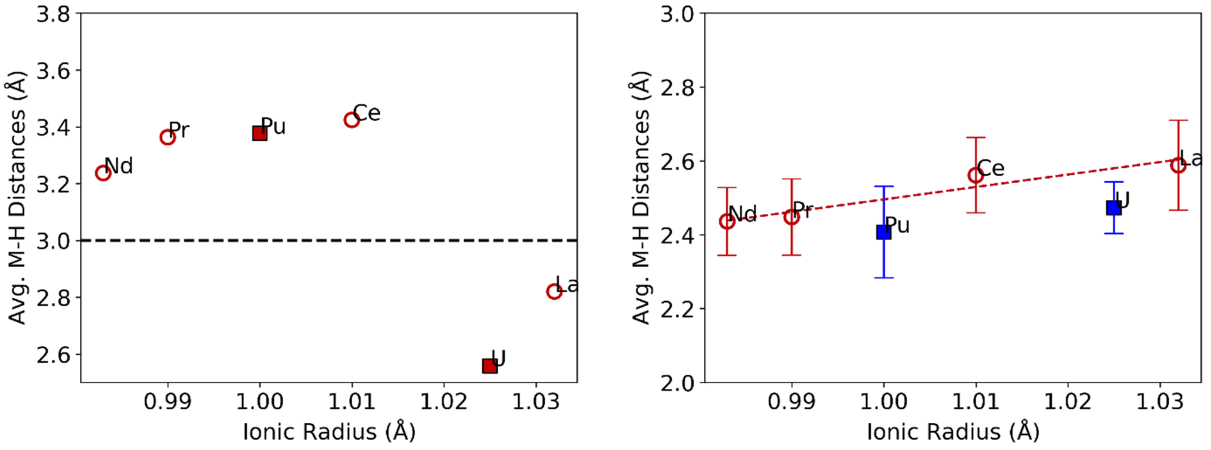

Figure S17. M-H bond distances for the bridging ligand. The $–BH_3$ groups on the bridging ligand can be κ^2 or κ^3. The plot on the left shows the longest M-H distance; therefore, one of the $–BH_3$ groups is κ^2 for the metals above the black dashed line while for the metals below the dashed line all $–BH_3$ groups are κ^3. Equivalent bonds on the two metal centers are averaged together. The plot on the right shows the average M-H distance including

only those shorter than 3.0 Å. Standard deviation is shown by the bars. Data for other metals from Ref [19]. Distances in Å.

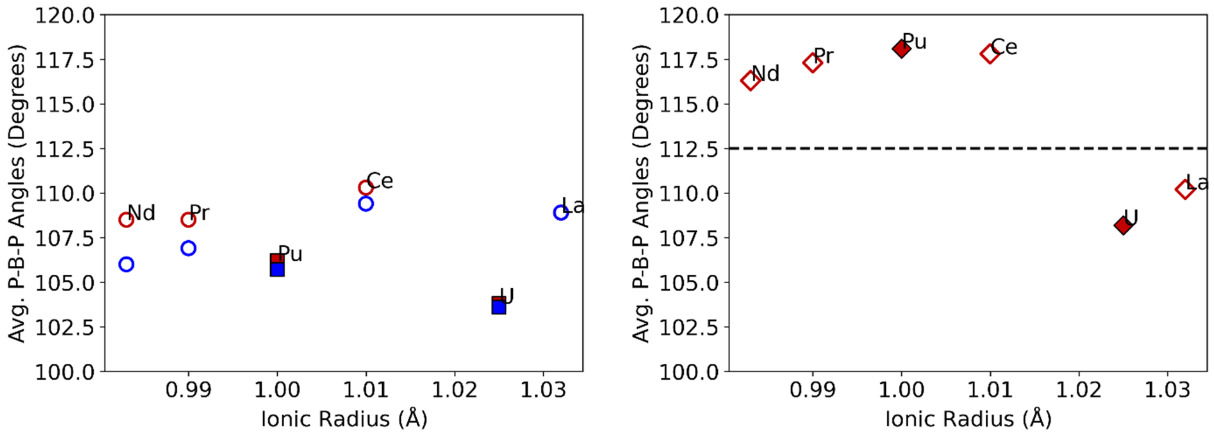

Figure S18. B-P-B angles for the chelating ligand (left) and the bridging ligand (right). The –BH_3 groups on the bridging ligand can be κ^2 or κ^3. The plot on the right shows a break denoted by the black dashed line between the metals where one of the –BH_3 groups is κ^2 (above the line) while for the metals below the dashed line all of the –BH_3 groups are κ^3. Equivalent angles on the two metal centers are averaged together. Data for other metals from Ref [19]. Angles in degrees.

5.2. Deoligimerization energies (TPSS-D3/def2-TZVP,def-TZVP)

Table S4. Calculated (TPSS-D3) thermochemical values for the dimer/monomer equilibrium observed for the trivalent plutonium complex. The corresponding experimental and calculated values previously reported for uranium and the lanthanides are included for comparison (taken from Ref [19]).

	**ΔH (kcal/mol)**		**ΔS (kcal/mol K)**		**ΔG (kcal/mol)**	
**Metal**	**Exp.**	**DFT**	**Exp.**	**DFT**	**Exp.**	**DFT**
U	10.5 ± 0.2	20.6	0.017 ± 0.001	0.054	5.3 ± 0.2	6.3
Pu	N/A	20.9	N/A	0.054	N/A	6.7
La	9.4 ± 0.6	18.6	0.016 ± 0.002	0.053	4.6 ± 0.6	4.8
Ce	8.9 ± 0.5	18.0	0.014 ± 0.002	0.053	4.7 ± 0.5	4.1
Pr	9.0 ± 0.4	18.6	0.015 ± 0.001	0.055	4.4 ± 0.4	4.2
Nd	8.7 ± 0.4	17.4	0.016 ± 0.001	0.053	4.0 ± 0.4	3.6

5.3. Bond Order Analysis (PBE/TZP)

Table S5. Nalewajski-Mrozek bond orders (PBE/TZP) for the trivalent actinide and lanthanide dimers for the An-B and Ln-B bonds.

**Metal**	**Ligand**	**La**	**Ce**	**Pr**	**Nd**	**U**	**Pu**
1	Chelating	0.244	0.345	0.192	0.197	0.438	0.163
		0.232	0.329	0.189	0.188	0.417	0.137
		0.233	0.346	0.176	0.190	0.421	0.173
		0.247	0.359	0.195	0.193	0.439	0.168
	Bridging	0.328	0.334	0.254	0.206	0.598	0.180
		0.300	0.458	0.196	0.275	0.645	0.224
2	Chelating	0.245	0.345	0.191	0.192	0.437	0.163
		0.232	0.329	0.188	0.188	0.417	0.137
		0.234	0.346	0.177	0.190	0.421	0.173
		0.247	0.359	0.195	0.199	0.439	0.168
	Bridging	0.299	0.458	0.195	0.272	0.645	0.223
		0.328	0.335	0.254	0.210	0.600	0.180

Table S6. Average chelating and bridging Nalewajski-Mrozek bond orders (PBE/TZP) for the trivalent actinide and lanthanide dimers for the An-B and Ln-B bonds. Standard deviations are included.

Metal	Chelating		Bridging	
	**Avg**	**St Dev**	**Avg**	**St Dev**
U	0.428	0.010	0.622	0.027
Pu	0.160	0.015	0.202	0.025
La	0.239	0.007	0.314	0.017
Ce	0.345	0.011	0.396	0.071
Pr	0.188	0.007	0.225	0.033
Nd	0.192	0.004	0.241	0.038

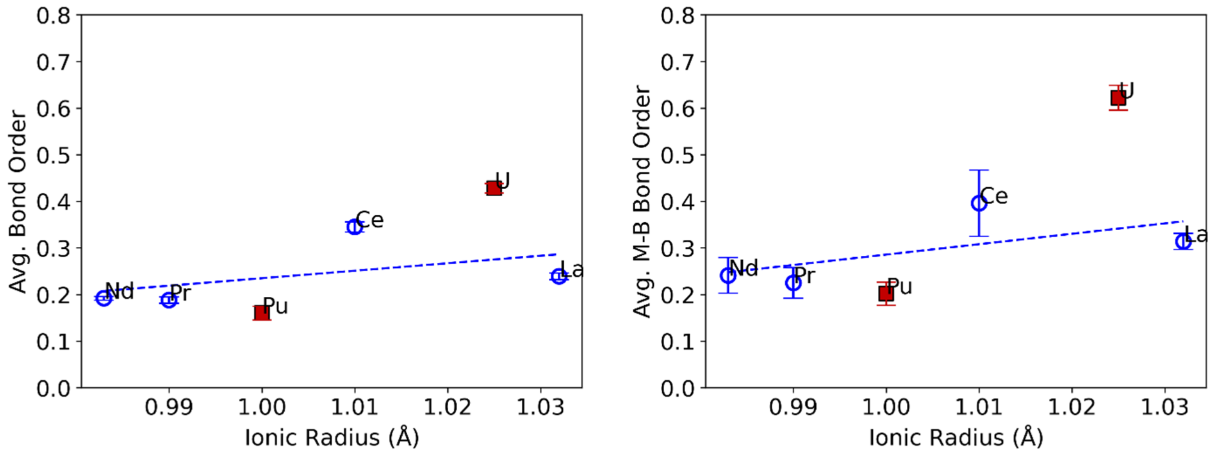

Figure S19. Average chelating (left) and bridging (right) Nalewajski-Mrozek bond orders (PBE/TZP) for the trivalent actinide and lanthanide dimers for the An-B and Ln-B bonds. Averages over all bond distances of each type. Standard deviations are included as error bars.

Table S7. Chelating and bridging Nalewajski-Mrozek bond orders (PBE/TZP) for the trivalent actinide and lanthanide dimers for the An-H and Ln-H bonds.

Metal Center	type	La	Ce	Pr	Nd	U	Pu
1	chelating	0.101	0.140	0.079	0.079	0.181	0.097
		0.114	0.162	0.096	0.098	0.229	0.067
		0.095	0.135	0.079	0.070	0.175	0.071
		0.107	0.152	0.089	0.093	0.211	0.082
		0.093	0.134	0.065	0.080	0.173	0.051
		0.109	0.160	0.086	0.091	0.214	0.101
		0.101	0.145	0.078	0.077	0.189	
		0.115	0.166	0.096	0.101	0.220	
	bridging	0.088	0.168	0.063	0.097	0.187	0.086
		0.099	0.137	0.066	0.080	0.193	0.073
		0.095	0.116	0.094	0.075	0.155	0.070
		0.056	0.125	0.091	0.077	0.159	0.082
		0.113	0.171	0.083	0.092	0.217	
		0.090				0.206	
**2**	chelating	0.101	0.140	0.079	0.068	0.181	0.097
		0.114	0.162	0.096	0.101	0.228	0.067
		0.094	0.135	0.078	0.072	0.176	0.070
		0.107	0.152	0.090	0.091	0.211	0.082
		0.093	0.134	0.065	0.075	0.173	0.051
		0.109	0.160	0.086	0.096	0.214	0.101
		0.101	0.144	0.078	0.085	0.188	
		0.115	0.166	0.096	0.100	0.220	
	bridging	0.056	0.116	0.091	0.072	0.159	0.070
		0.114	0.124	0.082	0.075	0.215	0.082
		0.089	0.171	0.063	0.094	0.207	0.086

0.087	0.168	0.067	0.099	0.187	0.073
0.099	0.137	0.093	0.082	0.193	0.097
0.096	0.140	0.079	0.079	0.156	0.067

Table S8. Average chelating and bridging Nalewajski-Mrozek bond orders (PBE/TZP) for the trivalent actinide and lanthanide dimers for the An-H and Ln-H bonds. Standard deviations are included.

Metal	Chelating		Bridging	
	Avg	St Dev	Avg	St Dev
U	0.199	0.021	0.186	0.023
Pu	0.078	0.018	0.078	0.007
La	0.104	0.008	0.090	0.018
Ce	0.149	0.012	0.143	0.024
Pr	0.083	0.010	0.079	0.013
Nd	0.086	0.012	0.084	0.010

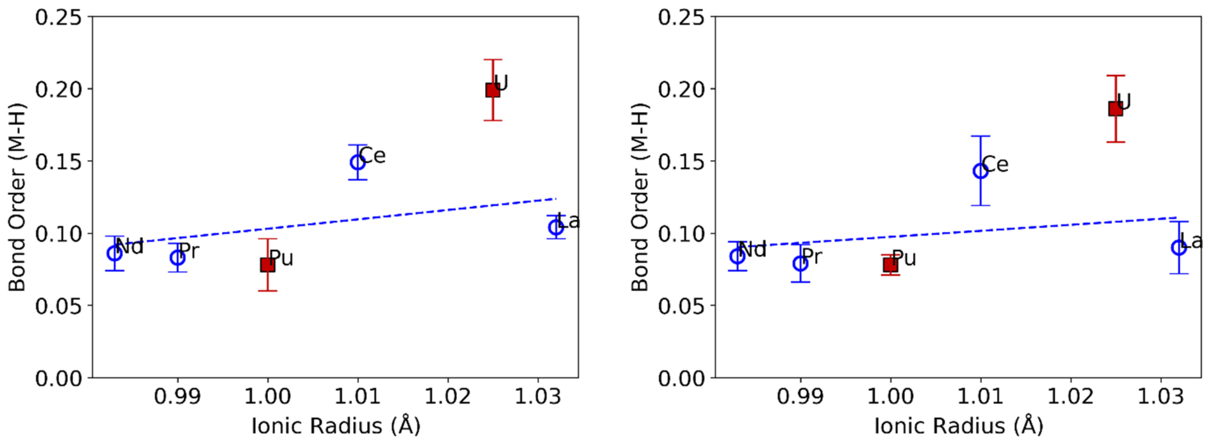

Figure S20. Average chelating (left) and bridging (right) Nalewajski-Mrozek bond orders (PBE/TZP) for the trivalent actinide and lanthanide dimers for the An-H and Ln-H bonds. Standard deviations are included as error bars.

Table S9. Sum per ligand (4 chelating vs 2 bridging) of the chelating and bridging Nalewajski-Mrozek bond orders (PBE/TZP) for the trivalent actinide and lanthanide dimers for the An-H and Ln-H bonds. Standard deviations are included.

**Metal**	**Chelating**	**Bridging**
U	0.796	1.117
Pu	0.235	0.311
La	0.417	0.541
Ce	0.597	0.717
Pr	0.334	0.396
Nd	0.344	0.422

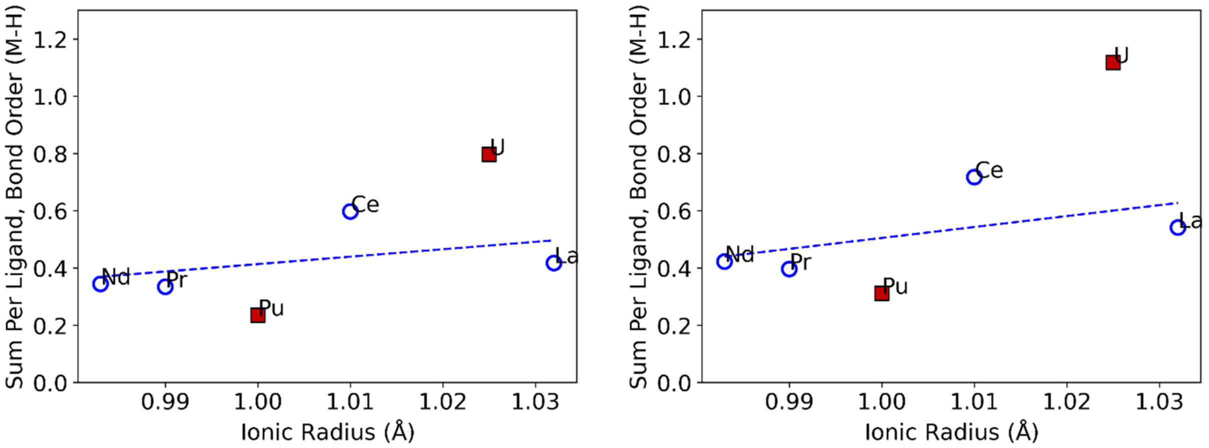

Figure S21. Sum per ligand (4 chelating vs 2 bridging) of the chelating (left) and bridging (right) Nalewajski-Mrozek bond orders (PBE/TZP) for the trivalent actinide and lanthanide dimers for the An-H and Ln-H bonds.

5.3. Energy Decomposition Analysis (EDA) (TPSS-D3/def2-TZVP,def-TZVP)

Table S10. DFT (PBE/TZP) Energy decomposition analysis (EDA) for the chelating ligands in the trivalent lanthanide and actinide dimers. Energies in kcal/mol.

Metal	Total Int. E	Pauli	Electrostatic E	Orbitalic E	% Elect	% Orb
La	-127.34	81.4	-135.52	-73.22	64.9	35.1
Ce	-126.94	101.06	-142.94	-85.07	62.8	37.3
Pr	-122.53	97.96	-138.93	-81.56	63.9	37.0
Nd	-120.94	98.97	-137.85	-82.06	62.7	37.3
U	-123.27	114.25	-144.59	-92.94	60.9	39.1
Pu	-119.93	113.17	-144.49	-88.61	62.0	38.0

Table S11. DFT (PBE/TZP) Energy decomposition analysis (EDA) for the bridging ligands in the trivalent lanthanide and actinide dimers. Energies in kcal/mol.

Metal	Total Int. E	Pauli	Elect. E	Orbitalic E	% Elect	% Orb
La	-122.66	111.86	-150.57	-83.95	64.2	35.8
Ce	-115.21	126.76	-156.35	-85.61	64.6	35.4
Pr	-116.20	132.15	-158.73	-89.61	63.9	36.1
Nd	-114.24	133.81	-159.39	-88.66	64.3	35.7
U	-127.27	170.41	-178.78	-118.90	60.1	39.9
Pu	-121.79	162.00	-179.84	-103.95	63.4	36.6

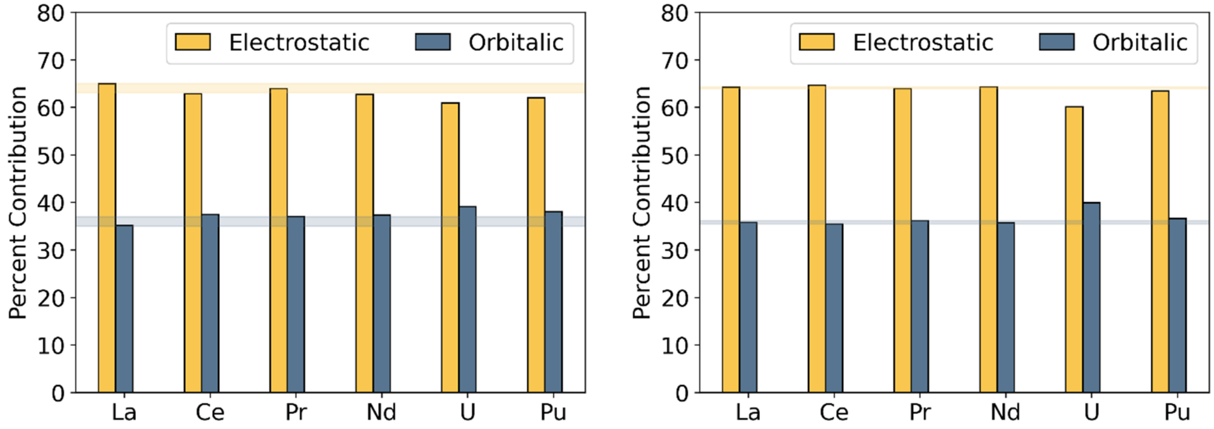

Figure S22. DFT (PBE/TZP) energy decomposition analysis (EDA) for the chelating (left) and bridging (right) ligands in the trivalent lanthanide and actinide dimers. Energies in kcal/mol. A horizontal region is shaded in highlighting the average value for the trivalent lanthanide ions, plus or minus the standard deviation.

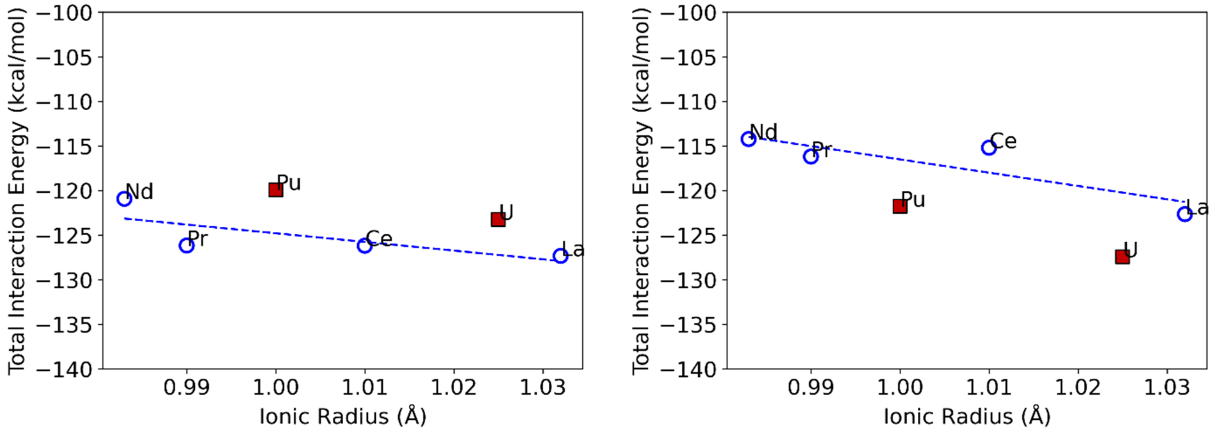

Figure S23. The total interaction energy (kcal/mol) from the DFT (PBE/TZP) energy decomposition analysis (EDA) for the chelating (left) and bridging (right) ligands in the trivalent lanthanide and actinide dimers. In all the lanthanides, the total interaction is STRONGER in chelating than bridging. But uranium, the chelating is weaker than bridging. Similar energies in Pu.

5.4. Delocalization Indices (TPSS-D3/def2-TZVP,def-TZVP)

Table S12. M-B delocalization indices (TPSS-D3) for the chelating and bridging ligands in the trivalent lanthanide and actinide dimers.

Metal Center	Ligand	La	Ce	Pr	Nd	U	Pu
**1**	chelating	0.058	0.071	0.069	0.064	0.075	0.064
		0.058	0.074	0.070	0.069	0.077	0.078
		0.061	0.074	0.073	0.069	0.080	0.082
		0.062	0.077	0.077	0.071	0.080	0.082
	bridging	0.079	0.072	0.073	0.075	0.113	0.082
		0.084	0.100	0.101	0.099	0.131	0.114
**2**	chelating	0.057	0.070	0.069	0.064	0.078	0.064
		0.060	0.073	0.069	0.069	0.077	0.078
		0.061	0.075	0.073	0.071	0.080	0.082
		0.062	0.076	0.076	0.072	0.079	0.083
	bridging	0.079	0.070	0.075	0.073	0.113	0.082
		0.087	0.099	0.102	0.104	0.127	0.114

Table S13. Average M-B delocalization indices (TPSS) for the chelating and bridging ligands in the trivalent lanthanide and actinide dimers. Data for other metals taken from Ref. [19].

Metal	Chelating		Bridging	
	**Avg DI**	**St Dev**	**Avg DI**	**St Dev**
U	0.078	0.002	0.121	0.009
Pu	0.077	0.008	0.098	0.018
La	0.060	0.002	0.082	0.004
Ce	0.074	0.002	0.085	0.016
Pr	0.072	0.003	0.088	0.016
Nd	0.069	0.003	0.088	0.016

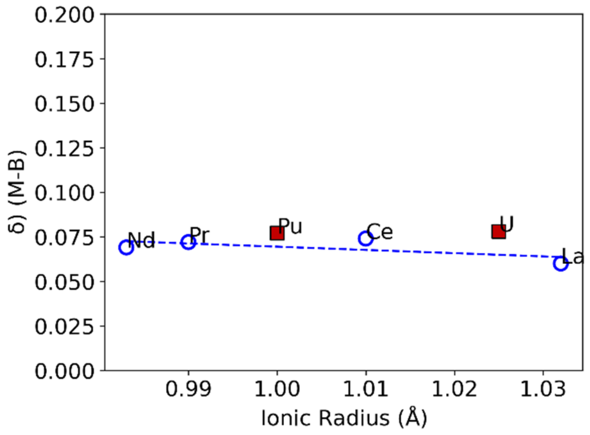

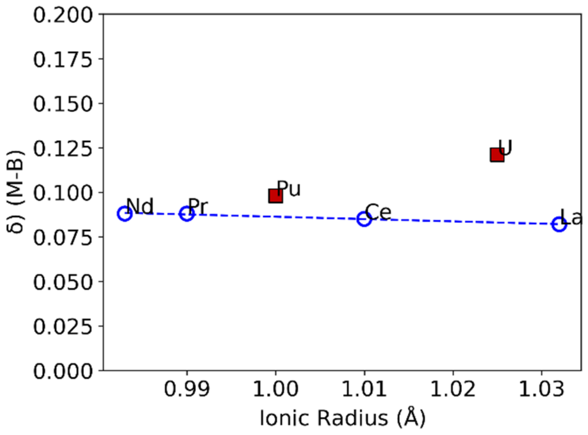

Figure S24. M-B delocalization indices (TPSS) for the chelating (left) and bridging (right) ligands in the trivalent lanthanide and actinide dimers. Data for other metals taken from Ref. [19].

5.5. Topological Analysis of the Electron Density, Quantum Theory of Atoms in Molecules (QTAIM) (TPSS-D3/def2-TZVP,def-TZVP)

Table S13. QTAIM (3,-1) bond critical points located between the Pu and B centers. Topological analysis is based on the TPSS-D3 density computed in Turbomole. Analysis performed with MultiWFN.

Pu Atom	B Atom	Ligand	ρ	$\nabla^2\rho$	V(r)	G(r)	E(r)
Pu 211	B 148	chelating	0.03393	0.08939	-0.02772	0.02503	-0.00269
Pu 211	B 156	chelating	No CP				
Pu 211	B 164	chelating	0.03663	0.09701	-0.03086	0.02756	-0.00330
Pu 211	B172	chelating	No CP				
Pu 212	B 147	chelating	0.03393	0.08937	-0.02772	0.02504	-0.00268
Pu 212	B 155	chelating	0.00630	0.01793	-0.00306	0.00377	0.00071
Pu 212	B 163	chelating	0.00730	0.02193	-0.00373	0.00460	0.00088
Pu 212	B 171	chelating	No CP				
Pu 211	B 187	bridging	0.04612	0.11345	-0.04231	0.03534	-0.00697
Pu 211	B 180	bridging	No CP				
Pu 212	B 188	bridging	0.04610	0.11341	-0.04230	0.03533	-0.00697
Pu 212	B179	bridging	No CP				

Table S14. Average parameters from QTAIM for the bond critical points that were found located between the Pu and B centers in the Pu dimer. Topological analysis is based on the TPSS-D3 density computed in Turbomole. Analysis performed with MultiWFN.

Metal	Ligand Type	ρ	$\nabla^2\rho$	V(r)	G(r)	E(r)
Pu	chelating	0.03528	0.09320	-0.02929	0.02630	-0.00300
	bridging	0.04611	0.11343	-0.04231	0.03534	-0.00697

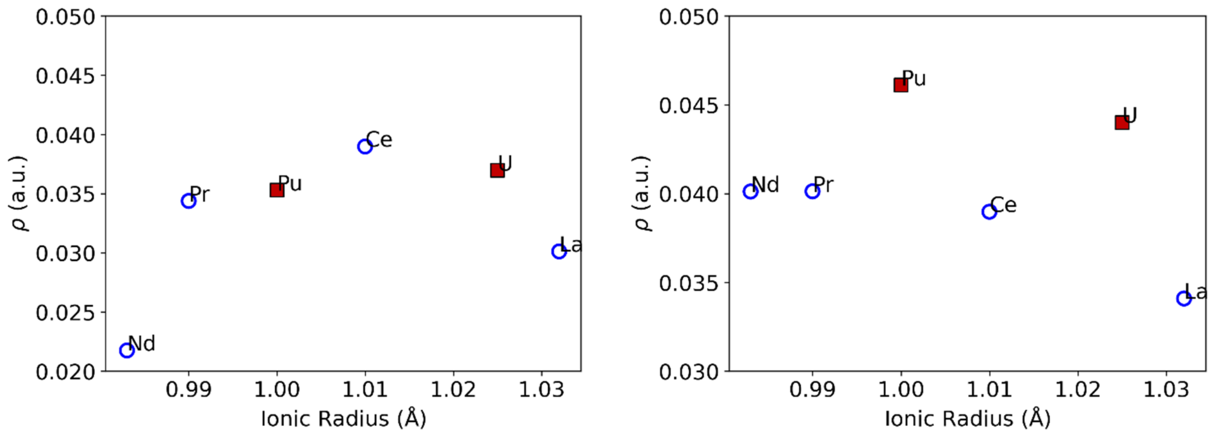

Figure S25. Average QTAIM electron density at the M-B bond critical points (TPSS-D3) for the chelating (left) and bridging (right) ligands in the trivalent lanthanide and actinide dimers. Data for other metals taken from Ref. [19].

5.6. Topological Analysis of the Electron Density, Quantum Theory of Atoms in Molecules (QTAIM) (PBE/TZP)

Table S15. QTAIM (3,-1) bond critical points located between the Pu and H centers. Topological analysis is based on the PBE density computed in ADF.

Metal	Ligand	ρ	∇2ρ	V(r)	G(r)	E(r)
1	chelating	0.03706	0.10194	-0.03215	0.02882	-0.00333
	chelating	0.03994	0.10542	-0.03558	0.03097	-0.00461
	chelating	0.04153	0.10974	-0.03775	0.03259	-0.00516
	chelating	No CP				
2	chelating	0.03566	0.09298	-0.02993	0.02659	-0.00334
	chelating	No CP				
	chelating	0.04051	0.10562	-0.03624	0.03133	-0.00492
	chelating	No CP				
1	bridging	0.04416	0.11286	-0.04109	0.03465	-0.00644
	bridging	0.04631	0.11997	-0.04429	0.03714	-0.00715
2	bridging	0.04632	0.12000	-0.04430	0.03715	-0.00715
	bridging	0.04418	0.11290	-0.04112	0.03467	-0.00645

Table S16. Average parameters from QTAIM for the bond critical points that were found located between the M and H centers in the dimers. Topological analysis is based on the PBE density computed in ADF.

Metal	Ligand	ρ	∇2ρ	V(r)	G(r)	E(r)
U	chelating	0.03690	0.09980	-0.03185	0.02840	-0.00345
	bridging	0.04457	0.11096	-0.04145	0.03459	-0.00685
Pu	chelating	0.03915	0.10328	-0.03458	0.03020	-0.00438
	bridging	0.04524	0.11643	-0.04270	0.03590	-0.00680
La	chelating	0.03076	0.07860	-0.02390	0.02178	-0.00213
	bridging	0.03466	0.09074	-0.02872	0.02570	-0.00302
Ce	chelating	0.03363	0.08749	-0.02743	0.02465	-0.00278
	bridging	0.03744	0.09544	-0.03203	0.02795	-0.00409
Pr	chelating	0.03398	0.08982	-0.02797	0.02521	-0.00276
	bridging	0.03872	0.09911	-0.03373	0.02925	-0.00448
Nd	chelating	0.03394	0.09111	-0.02804	0.02541	-0.00263
	bridging	0.03903	0.10090	-0.03421	0.02972	-0.00449

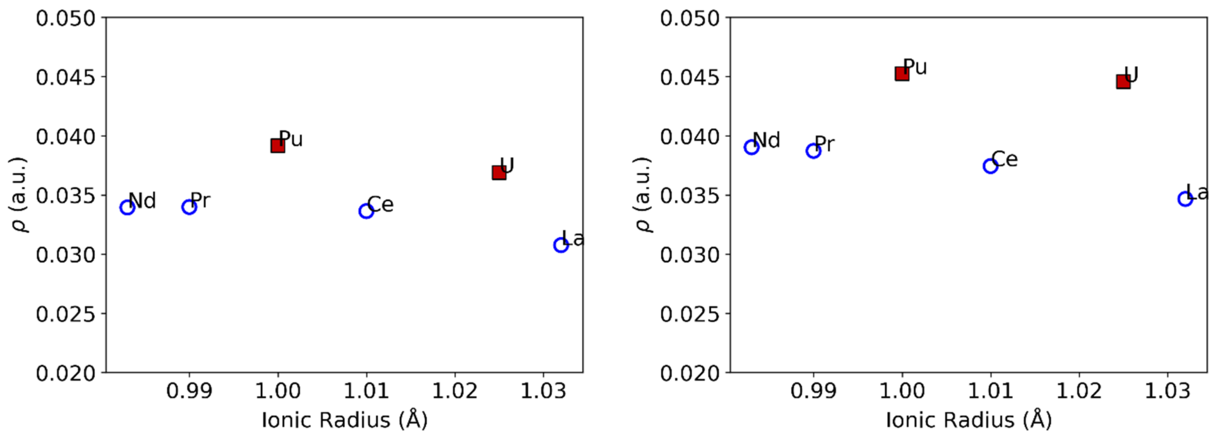

Figure S26. Average QTAIM electron density at the M-H bond critical points (PBE/TZVP) for the chelating (left) and bridging (right) ligands in the trivalent lanthanide and actinide dimers.

Table S17. M-B delocalization indices (PBE/TZP) for the chelating and bridging ligands in the trivalent lanthanide and actinide dimers.

Metal Center	Ligand	La	Ce	Pr	Nd	U	Pu
1	chelating	0.0729	0.0836	0.0806	0.0818	0.0916	0.0961
		0.0754	0.0847	0.0871	0.0822	0.0939	0.0859
		0.0746	0.0822	0.0829	0.0825	0.0962	0.0987
		0.0718	0.0774	0.0825	0.0807	0.0906	0.0998
	bridging	0.0946	0.1101	0.0842	0.0852	0.1482	0.1019
		0.0983	0.0774	0.1138	0.1170	0.1323	0.1342
**2**	chelating	0.0736	0.0815	0.0829	0.0824	0.0804	0.0965
		0.0728	0.0832	0.0803	0.0813	0.0766	0.0858
		0.0709	0.0772	0.0841	0.0802	0.0757	0.0988
		0.0760	0.0848	0.0868	0.0829	0.0794	0.0994
	bridging	0.0996	0.0769	0.1146	0.1154	0.1301	0.1344
		0.0957	0.1114	0.0833	0.0854	0.1482	0.1010

Table S18. M-B delocalization indices (PBE/TZP) for the chelating and bridging ligands in the trivalent lanthanide and actinide dimers. Standard deviations are also reported.

Metal	Chelating		Bridging	
	Avg DI	St Dev	Avg DI	St Dev
U	0.086	0.008	0.140	0.010
Pu	0.095	0.006	0.118	0.019
La	0.074	0.002	0.097	0.002
Ce	0.082	0.003	0.094	0.019
Pr	0.083	0.003	0.099	0.018
Nd	0.082	0.001	0.101	0.018

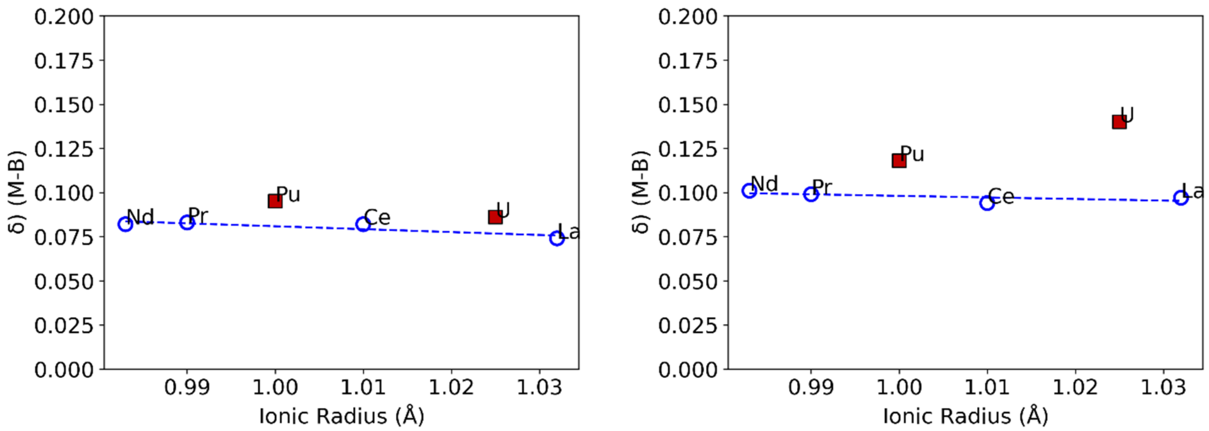

Figure S27. Average M-B delocalization indices (PBE/TZP) for the chelating (left) and bridging (right) ligands in the trivalent lanthanide and actinide dimers.

Table S19. M-H delocalization indices (PBE/TZP) for the chelating and bridging ligands in the trivalent lanthanide and actinide dimers.

Metal Center	type	La	Ce	Pr	Nd	U	Pu
**1**	chelating	0.1384	0.1478	0.1538	0.1503	0.1724	0.1608
		0.1539	0.1651	0.1734	0.1737	0.2143	0.2221
		0.0249	0.0266	0.0271	0.0281	0.0271	0.0303
	chelating	0.1262	0.1369	0.1422	0.1277	0.1645	0.1382
		0.1456	0.1570	0.1619	0.1619	0.2022	0.1755
		0.0255	0.0270	0.0267	0.0283	0.0272	0.0285
	chelating	0.1283	0.1432	0.1354	0.1451	0.1616	0.1840
		0.1490	0.1627	0.1674	0.1690	0.2102	0.2011
		0.0256	0.0280	0.0271	0.0274	0.0280	0.0296
	chelating	0.1365	0.1479	0.1529	0.1478	0.1773	0.1580
		0.1572	0.1735	0.1763	0.1754	0.2079	0.2269
		0.0250	0.0277	0.0278	0.0264	0.0272	0.0307
	bridging	0.1179	0.0194	0.1221	0.1312	0.1699	0.2289

		0.1364	0.1827	0.1331	0.1425	0.1783	0.1832
		0.1307	0.1384	0.1803	0.1722	0.1407	0.0255
	bridging	0.0737	0.1149	0.0208	0.0241	0.1451	0.1286
		0.1568	0.1293	0.1862	0.1856	0.1953	0.1737
		0.1209	0.1479	0.1471	0.1388	0.1924	0.2110
**2**	chelating	0.1276	0.1439	0.1352	0.1408	0.1617	0.0303
		0.1478	0.1620	0.1672	0.1707	0.2093	0.1608
		0.0254	0.0280	0.0270	0.0266	0.0279	0.2218
	chelating	0.1368	0.1475	0.1530	0.1512	0.1774	0.1384
		0.1572	0.1730	0.1768	0.1759	0.2087	0.1752
		0.0250	0.0275	0.0280	0.0270	0.0274	0.0284
	chelating	0.1270	0.1370	0.1422	0.1295	0.1632	0.1837
		0.1467	0.1569	0.1610	0.1597	0.2016	0.2010
		0.0254	0.0269	0.0268	0.0281	0.0272	0.0297
	chelating	0.1380	0.1476	0.1541	0.1436	0.1732	0.1580
		0.1539	0.1652	0.1729	0.1762	0.2159	0.2274
		0.0249	0.0266	0.0271	0.0273	0.0271	0.0307
	bridging	0.0743	0.1137	0.0217	0.0247	0.1463	0.1288
		0.1561	0.1295	0.1873	0.1861	0.1981	0.1734
		0.1221	0.1747	0.1490	0.1407	0.191	0.2110
	bridging	0.1175	0.0191	0.1229	0.1307	0.1711	0.2296
		0.1376	0.1827	0.1320	0.1401	0.1789	0.1828
		0.1305	0.1381	0.1800	0.1740	0.1403	0.0256

Table S20. Average M-H delocalization indices (PBE/TZP) for the chelating and bridging ligands in the trivalent lanthanide and actinide dimers. Only the coordinating hydrogen atoms per $-BH_3$ group are included in the average. Standard deviations are also reported.

**Metal**	**Chelating**		**Bridging**	
	**Avg DI**	**St Dev**	**Avg DI**	**St Dev**
U	0.189	0.021	0.171	0.022
Pu	0.183	0.030	0.185	0.036
La	0.142	0.011	0.123	0.026
Ce	0.154	0.012	0.145	0.026
Pr	0.158	0.014	0.154	0.027
Nd	0.156	0.016	0.154	0.022

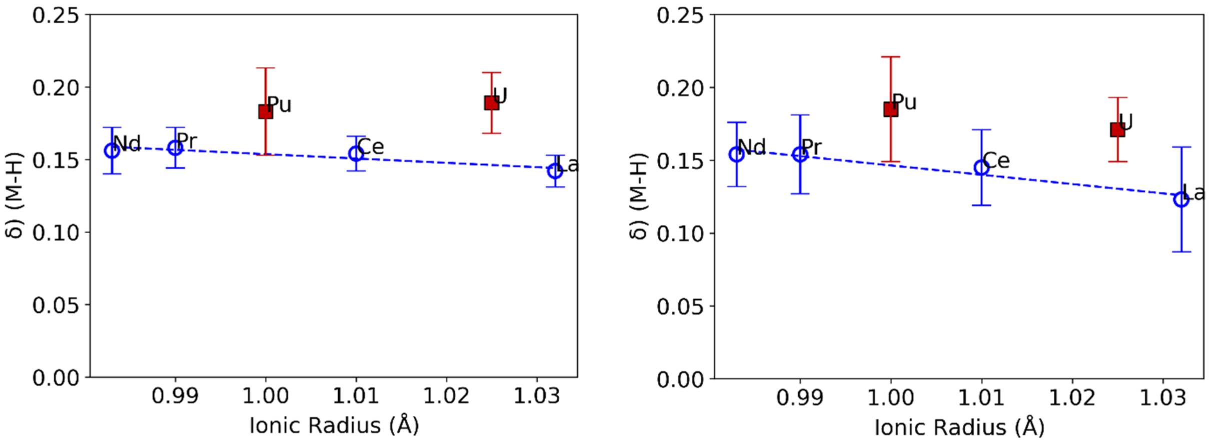

Figure S28. Average M-H delocalization indices (PBE/TZP) for the chelating (left) and bridging (right) ligands in the trivalent lanthanide and actinide dimers. Only the coordinating hydrogen atoms per $-BH_3$ group are included in the average. Standard deviations are also reported.

Table S21. Sum per ligand of the M-H delocalization indices (PBE/TZP) for the chelating and bridging ligands in the trivalent lanthanide and actinide dimers. All three hydrogen atoms are included in the average regardless of bond distance.

Metal	Chelating	Bridging
U	0.810	1.024
Pu	0.793	0.951
La	0.618	0.737
Ce	0.671	0.745
Pr	0.686	0.791
Nd	0.679	0.795

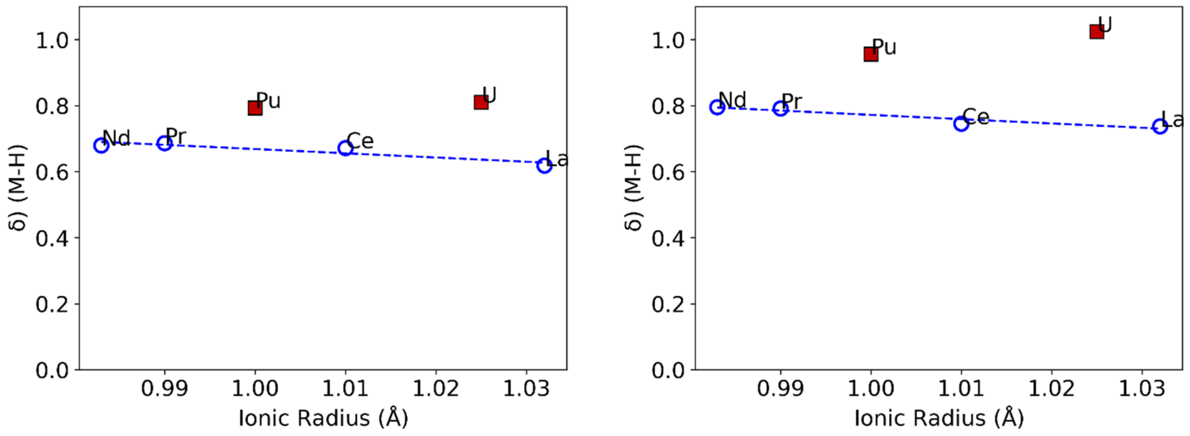

Figure S29. Sum per ligand of the M-H delocalization indices (PBE/TZP) for the chelating (left) and bridging (right) ligands in the trivalent lanthanide and actinide dimers.

Table S22. M-H delocalization indices (PBE/TZP) for the chelating and bridging ligands in the trivalent lanthanide and actinide dimers. Values are averaged for each of the three M-H interactions separately (each BH_3 group has three interactions of varying strength). For the bridging ligands, the third hydrogen is only coordinated in some cases. Therefore, the four individual values are averaged in two sets (shortest and longest) to emphasize this change in coordination number.

Type	Distance	U	Pu	La	Ce	Pr	Nd
chelating	longest	0.209	0.209	0.151	0.164	0.170	0.170
	middle	0.169	0.158	0.132	0.144	0.146	0.142
	uncoordinated	0.027	0.030	0.025	0.027	0.027	0.027
bridging	longest	0.194	0.220	0.147	0.172	0.183	0.179
	middle	0.175	0.178	0.126	0.134	0.140	0.141
	shortest	0.146	0.129	0.118	0.114	0.123	0.1310
		0.141	0.026	0.074	0.019	0.021	0.024

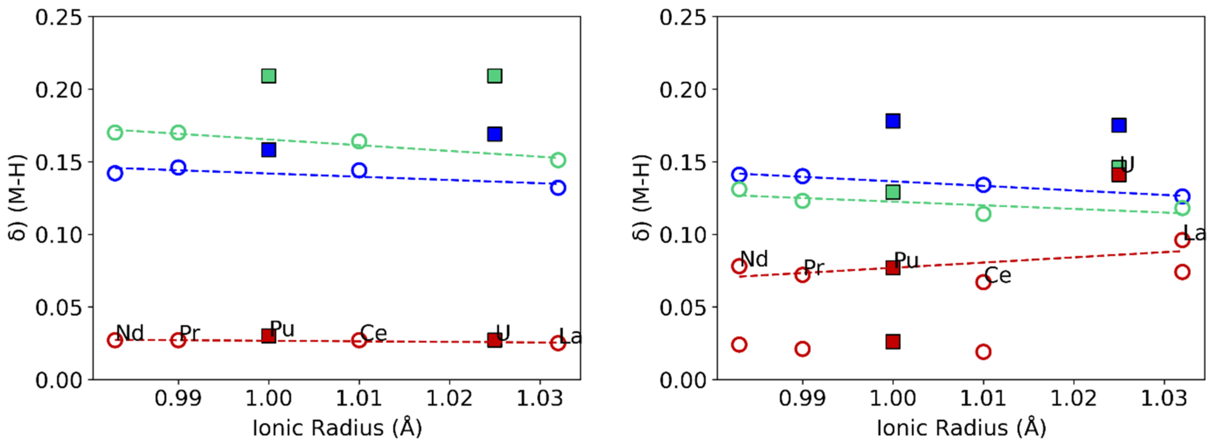

Figure S30. M-H delocalization indices (PBE/TZP) for the chelating (left) and bridging (right) ligands in the trivalent lanthanide and actinide dimers. Values are averaged for each of the three M-H interactions separately (each BH_3 group has three interactions of varying strength). For the bridging ligand, two points are plotted in red to emphasize the higher coordination number in U and La.

6. Supporting Information References

(1) Fetrow, T. V.; Grabow, J. P.; Leddy, J.; Daly, S. R. Convenient Syntheses of Trivalent Uranium Halide Starting Materials without Uranium Metal. *Inorg. Chem.* **2021**, *60*, 7593-7601.

(2) Dornhaus, F.; Bolte, M. (18-Crown-6)potassium di-tert-butylphosphanylbisborohydride. *Acta Crystallogr., Sect. E Struct. Rep. Online* **2006**, *62*, m3573-m3575.

(3) Goodwin, C. A. P.; Janicke, M. T.; Scott, B. L.; Gaunt, A. J. [$AnI_3(THF)_4$] (An = Np, Pu) Preparation Bypassing An^0 Metal Precursors: Access to Np^{3+}/Pu^{3+} Nonaqueous and Organometallic Complexes. *J. Am. Chem. Soc.* **2021**, *143*, 20680-20696.

(4) Wang, F.; Ramakrishna, S. K.; Sun, P.; Fu, R. Triple-pulse excitation: An efficient way for suppressing background signals and eliminating radio-frequency acoustic ringing in direct polarization NMR experiments. *Journal of Magnetic Resonance* **2021**, *332*, 107067.

(5) Fulmer, G. R.; Miller, A. J. M.; Sherden, N. H.; Gottlieb, H. E.; Nudelman, A.; Stoltz, B. M.; Bercaw, J. E.; Goldberg, K. I. NMR Chemical Shifts of Trace Impurities: Common Laboratory Solvents, Organics, and Gases in Deuterated Solvents Relevant to the Organometallic Chemist. *Organometallics* **2010**, *29*, 2176-2179.

(6) Blake, A. V.; Fetrow, T. V.; Theiler, Z. J.; Vlaisavljevich, B.; Daly, S. R. Homoleptic uranium and lanthanide phosphinodiboranates. *Chem. Commun.* **2018**, *54*, 5602-5605.

(7) APEX v2021.4.0, **2021**, Bruker AXS, Inc., Madison, Wisconsin 53719.

(8) SADABS v2016/2, **2016**, George Sheldrick, University of Göttingen, Germany.

(9) Spek, A. Single-crystal structure validation with the program PLATON. *J. Appl. Crystallogr.* **2003**, *36*, 7-13.

(10) Spek, A. L. PLATON SQUEEZE: a tool for the calculation of the disordered solvent contribution to the calculated structure factors. *Acta Crystallogr., Sect. C Struct. Chem.* **2015**, *71*, 9-18.

(11) SHELXTL 2018/3, **2018**, George Sheldrick, University of Göttingen, Germany.

(12) Macrae, C. F.; Edgington, P. R.; McCabe, P.; Pidcock, E.; Shields, G. P.; Taylor, R.; Towler, M.; van de Streek, J. Mercury: visualization and analysis of crystal structures. *J. Appl. Crystallogr.* **2006**, *39*, 453-457.

(13) Parsons, S.; Clegg, W. Random and systematic errors. In *Crystal Structure Analysis: Principles and Practice*; Oxford University Press, 2009; pp 221-250. DOI: 10.1093/acprof:oso/9780199219469.003.0016.

(14) Apostolidis, C.; Schimmelpfennig, B.; Magnani, N.; Lindqvist-Reis, P.; Walter, O.; Sykora, R.; Morgenstern, A.; Colineau, E.; Caciuffo, R.; Klenze, R.; Haire, R. G.; Rebizant, J.; Bruchertseifer, F.; Fanghänel, T. $[An(H_2O)_9](CF_3SO_3)_3$ (An=U–Cm, Cf): Exploring Their Stability, Structural Chemistry, and Magnetic Behavior by Experiment and Theory. *Angewandte Chemie International Edition* **2010**, *49*, 6343-6347.

(15) Polinski, M. J.; Wang, S.; Alekseev, E. V.; Depmeier, W.; Albrecht-Schmitt, T. E. Bonding Changes in Plutonium(III) and Americium(III) Borates. *Angewandte Chemie International Edition* **2011**, *50*, 8891-8894.

(16) Jones, M. B.; Gaunt, A. J.; Gordon, J. C.; Kaltsoyannis, N.; Neu, M. P.; Scott, B. L. Uncovering f-element bonding differences and electronic structure in a series of 1:3 and 1:4 complexes with a diselenophosphinate ligand. *Chem. Sci.* **2013**, *4*, 1189-1203.

(17) Cross, J. N.; Cary, S. K.; Stritzinger, J. T.; Polinski, M. J.; Albrecht-Schmitt, T. E. Synthesis and Spectroscopy of New Plutonium(III) and -(IV) Molybdates: Comparisons of Electronic Characteristics. *Inorg. Chem.* **2014**, *53*, 3148-3152.

(18) Goodwin, C. A. P.; Ciccone, S. R.; Bekoe, S.; Majumdar, S.; Scott, B. L.; Ziller, J. W.; Gaunt, A. J.; Furche, F.; Evans, W. J. 2.2.2-Cryptand complexes of neptunium(III) and plutonium(III). *Chem. Commun.* **2022**, *58*, 997-1000.

(19) Fetrow, T. V.; Zgrabik, J.; Bhowmick, R.; Eckstrom, F. D.; Crull, G.; Vlaisavljevich, B.; Daly, S. R. Quantifying the Influence of Covalent Metal-Ligand Bonding on Differing Reactivity of Trivalent Uranium and Lanthanide Complexes. *Angew. Chem., Int. Ed.* **2022**, *61*, e202211145.

(20) Tao, J.; Perdew, J. P.; Staroverov, V. N.; Scuseria, G. E. Climbing the density functional ladder: nonempirical meta-generalized gradient approximation designed for molecules and solids. *Phys. Rev. Lett.* **2003**, *91*, 146401/146401-146401/146404.

(21) Grimme, S.; Antony, J.; Ehrlich, S.; Krieg, H. A consistent and accurate ab initio parametrization of density functional dispersion correction (DFT-D) for the 94 elements H-Pu. *J. Chem. Phys.* **2010**, *132*, 154104/154101-154104/154119.

(22) Eichkorn, K.; Treutler, O.; Oehm, H.; Haeser, M.; Ahlrichs, R. Auxiliary basis sets to approximate Coulomb potentials. [Erratum to document cited in CA123:93649]. *Chem. Phys. Lett.* **1995**, *242*, 652-660.

(23) Eichkorn, K.; Weigend, F.; Treutler, O.; Ahlrichs, R. Auxiliary basis sets for main row atoms and transition metals and their use to approximate Coulomb potentials. *Theor. Chem. Acc.* **1997**, *97*, 119-124.

(24) Dolg, M.; Stoll, H.; Preuss, H. Energy-adjusted ab initio pseudopotentials for the rare earth elements. *J. Chem. Phys.* **1989**, *90*, 1730-1734.

(25) Dolg, M.; Stoll, H.; Preuss, H. A combination of quasirelativistic pseudopotential and ligand field calculations for lanthanoid compounds. *Theor. Chim. Acta* **1993**, *85*, 441-450.

(26) Kuechle, W.; Dolg, M.; Stoll, H.; Preuss, H. Energy-adjusted pseudopotentials for the actinides. Parameter sets and test calculations for thorium and thorium monoxide. *J. Chem. Phys.* **1994**, *100*, 7535-7542.

(27) Cao, X.; Dolg, M. Valence basis sets for relativistic energy-consistent small-core lanthanide pseudopotentials. *J. Chem. Phys.* **2001**, *115*, 7348-7355.

(28) Cao, X.; Dolg, M.; Stoll, H. Valence basis sets for relativistic energy-consistent small-core actinide pseudopotentials. *J. Chem. Phys.* **2003**, *118*, 487-496.

(29) Weigend, F.; Haser, M.; Patzelt, H.; Ahlrichs, R. RI-MP2: optimized auxiliary basis sets and demonstration of efficiency. *Chem. Phys. Lett.* **1998**, *294*, 143-152.

(30) Klamt, A.; Schueuermann, G. COSMO: a new approach to dielectric screening in solvents with explicit expressions for the screening energy and its gradient. *J. Chem. Soc., Perkin Trans. 2* **1993**, 799-805.

(31) R. Ahlrichs, M. Bär, H.-P. Baron, R. Bauernschmitt, S. Böcker, N. Crawford, P. Deglmann, M. Ehrig, K. Eichkorn, S. Elliott, F. Furche, F. Haase, M. Häser, C. Hättig, A. Hellweg, H. Horn, C. Huber, U. Huniar, M. Kattannek, A. Köhn, C. Kölmel, M. Kollwitz, K. May, P. Nava, C. Ochsenfeld, H. Öhm, H. Patzelt, D. Rappoport, O. Rubner, A. Schäfer, U. Schneider, M. Sierka, O. Treutler, B. Unterreiner, M. von Arnim, F. Weigend, P. Weis, and H. Weiss, *Turbomole, version 7.3*, Universität Karlsruhe, 2018.

(32) Ribeiro, R. F.; Marenich, A. V.; Cramer, C. J.; Truhlar, D. G. Use of Solution-Phase Vibrational Frequencies in Continuum Models for the Free Energy of Solvation. *J. Phys. Chem. B* **2011**, *115*, 14556-14562.

(33) Bader, R. F. W. A quantum theory of molecular structure and its applications. *Chem. Rev.* **1991**, *91*, 893-928.

(34) te Velde, G.; Bickelhaupt, F. M.; Baerends, E. J.; Fonseca Guerra, C.; van Gisbergen, S. J. A.; Snijders, J. G.; Ziegler, T. Chemistry with ADF. *J. Comput. Chem.* **2001**, *22*, 931-967.

(35) Perdew, J. P.; Burke, K.; Ernzerhof, M. Generalized gradient approximation made simple. *Phys. Rev. Lett.* **1996**, *77*, 3865-3868.

(36) Perdew, J. P.; Burke, K.; Ernzerhof, M. Generalized gradient approximation made simple. [Erratum to document cited in CA126:51093]. *Phys. Rev. Lett.* **1997**, *78*, 1396.

(37) van Lenthe, E.; Baerends, E. J.; Snijders, J. G. Relativistic total energy using regular approximations. *The Journal of Chemical Physics* **1994**, *101*, 9783-9792.

(38) Lu, T.; Chen, F. Multiwfn: A multifunctional wavefunction analyzer. *J. Comput. Chem.* **2012**, *33*, 580-592.
